# Tumour Microenvironments Induce Expression of Urokinase Plasminogen Activator Receptor (uPAR) and Concomitant Activation of Gelatinolytic Enzymes

**DOI:** 10.1371/journal.pone.0105929

**Published:** 2014-08-26

**Authors:** Synnøve Magnussen, Elin Hadler-Olsen, Nadezhda Latysheva, Emma Pirila, Sonja E. Steigen, Robert Hanes, Tuula Salo, Jan-Olof Winberg, Lars Uhlin-Hansen, Gunbjørg Svineng

**Affiliations:** 1 Department of Medical Biology, Faculty of Health Sciences, UiT - The Arctic University of Norway, Tromsø, Norway; 2 Department of Diagnostics and Oral Medicine, Institute of Dentistry, University of Oulu, and Medical Research Center, Oulu University Hospital, Oulu, Finland; 3 Institute of Dentistry, University of Helsinki, Helsinki, Finland; 4 Diagnostic Clinic - Department of Clinical Pathology, University Hospital of North Norway, Tromsø, Norway; University of Patras, Greece

## Abstract

**Background:**

The urokinase plasminogen activator receptor (uPAR) is associated with poor prognosis in oral squamous cell carcinoma (OSCC), and increased expression of uPAR is often found at the invasive tumour front. The aim of the current study was to elucidate the role of uPAR in invasion and metastasis of OSCC, and the effects of various tumour microenvironments in these processes. Furthermore, we wanted to study whether the cells’ expression level of uPAR affected the activity of gelatinolytic enzymes.

**Methods:**

The *Plaur* gene was both overexpressed and knocked-down in the murine OSCC cell line AT84. Tongue and skin tumours were established in syngeneic mice, and cells were also studied in an *ex vivo* leiomyoma invasion model. Soluble factors derived from leiomyoma tissue, as well as purified extracellular matrix (ECM) proteins, were assessed for their ability to affect uPAR expression, glycosylation and cleavage. Activity of gelatinolytic enzymes in the tissues were assessed by *in situ* zymography.

**Results:**

We found that increased levels of uPAR did not induce tumour invasion or metastasis. However, cells expressing low endogenous levels of uPAR *in vitro* up-regulated uPAR expression both in tongue, skin and leiomyoma tissue. Various ECM proteins had no effect on uPAR expression, while soluble factors originating from the leiomyoma tissue increased both the expression and glycosylation of uPAR, and possibly also affected the proteolytic processing of uPAR. Tumours with high levels of uPAR, as well as cells invading leiomyoma tissue with up-regulated uPAR expression, all displayed enhanced activity of gelatinolytic enzymes.

**Conclusions:**

Although high levels of uPAR are not sufficient to induce invasion and metastasis, the activity of gelatinolytic enzymes was increased. Furthermore, several tumour microenvironments have the capacity to induce up-regulation of uPAR expression, and soluble factors in the tumour microenvironment may have an important role in the regulation of posttranslational modification of uPAR.

## Introduction

Oral squamous cell carcinoma (OSCC) is the most common malignancy of the oral cavity [1], [2], with a poor 5-year survival rate [2]–[4]. Urokinase-type plasminogen activator (uPA), a member of the plasminogen activation (PA) system, and its receptor, the urokinase plasminogen activator receptor (uPAR), have both been linked to poor prognosis in several cancer types [5]–[7], including OSCC [8]–[10]. The PA system consists of plasminogen which is the precursor of the active serine protease plasmin, its two activators (tissue-type plasminogen activator (tPA) and uPA), uPAR, as well as the inhibitors plasminogen activator inhibitor-1 (PAI-1) and PAI-2. uPA is secreted in its inactive pro-form (pro-uPA), and is readily activated in a feed-back-loop by plasmin upon binding to uPAR. uPAR is a highly glycosylated protein consisting of three homologous domains (D1, D2, and D3) and is linked to the plasma membrane via a GPI-anchor [11]. Plasmin functions as a broad spectrum protease that is able to degrade several extracellular matrix (ECM) proteins including gelatin [12], and activate latent growth factors and matrix metalloproteases (MMPs) [13]. Furthermore, plasmin, uPA, trypsin, chymotrypsin, cathepsin G, elastase and some MMPs are all able to cleave uPAR in the linker region between D1 and D2 [14]–[17]. This disrupts the receptor’s ability to bind uPA [18] in what is thought to be a natural regulation of the uPA-mediated proteolytic activity [19]. Cleavage of human uPAR can also expose the chemotactic SRSRY peptide (uPAR_88–92_) residing between D1 and D2 [20]. The SRSRY peptide can interact with the N-formyl peptide receptor (FPR), FPR-like 1 (FPRL1) and FPRL2 leading to directional cell migration [21]–[23]. Lastly, the GPI-anchor of uPAR may be cleaved by several phospholipases, releasing the soluble form of uPAR (suPAR), but also soluble uPAR D2+D3 either with or without the SRSRY peptide [17], [19], [24]–[26]. SuPAR and soluble cleaved forms of uPAR detected in either tissue and biological fluids may indicate an active PA-system and have been associated with poor prognosis in soft-tissue sarcoma, breast-, colorectal-, lung-, ovarian- and prostate cancer [27]–[38].

We previously observed that low expression of uPAR is associated with a favourable outcome in early stage OSCC [10]. Therefore, in the current study we wanted to elucidate the role of uPAR in invasive and metastatic tumour growth, and furthermore study how the tumour microenvironment participates in this process. To this end, tongue and skin tumours were established of the mouse OSCC cell line AT84 expressing either low uPAR levels or over-expressing uPAR. The cells were also analysed as they invaded the tissue of the leiomyoma invasion model [39]. Increased levels of uPAR did not lead to increased invasion or metastasis of these cells. However, the endogenous expression of uPAR was up-regulated in the initially low-uPAR expressing cells at the tumour stroma border *in vivo,* and as they invaded deep into the leiomyoma tissue. Analysis of gelatinolytic activity revealed that cells expressing high uPAR levels had an increased ability to activate gelatinolytic enzymes. When cells were stimulated *in vitro* with soluble factors derived from the leiomyoma stroma, an increase in the apparent molecular weight of the uPAR protein was observed, possibly due to increased glycosylation and/or an alteration in uPAR cleavage.

Together these results show that the tumour microenvironment can affect both the expression and posttranslational modifications of uPAR in the tumour cells, and thereby influence the activity of the gelatinolytic enzymes.

## Results

### Overexpression of uPAR in the murine AT84 cell line

**Figure 1:**
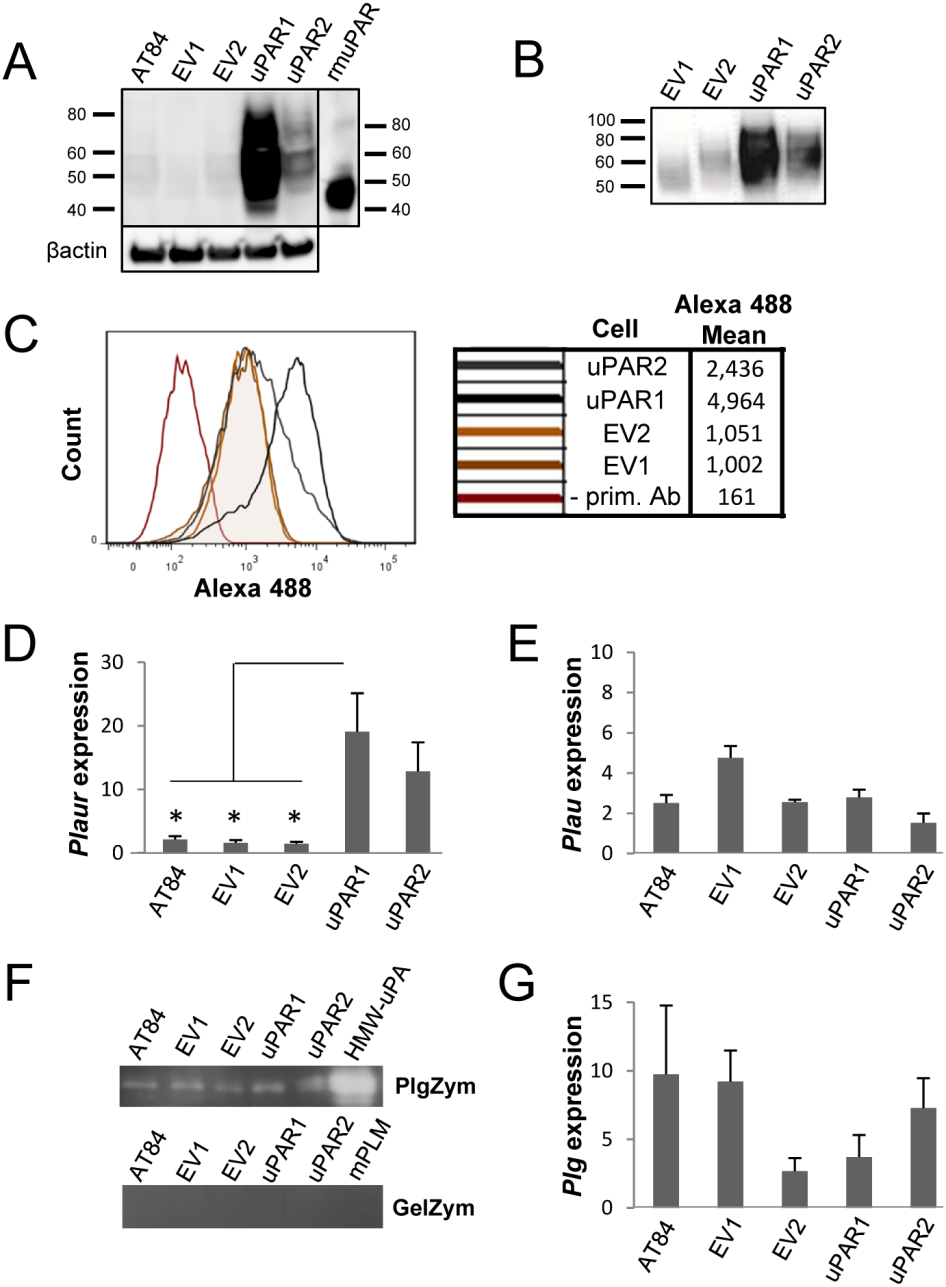
Expression of murine *Plaur* in AT84 cells. *In vitro* characterization of AT84 cells stably transfected with either empty vector (EV) or a vector containing cDNA encoding murine uPAR (*Plaur*). **A:** Western blot analysis of whole cell lysates using a polyclonal anti-murine uPAR antibody (AF534). A total of 7.5 ng of recombinant murine uPAR (rmuPAR) was loaded as a positive control. Re-probing for β-actin was used as a loading control. **B:** Western blot analysis of cellular membrane fractions using a polyclonal anti-murine uPAR antibody (AF534). Total protein was measured per sample and 53.5 µg of protein was loaded per lane. **A and B:** Images were cropped, as no additional bands were detectable. **C:** FACS analysis of non-permeabilized cells using a polyclonal anti-murine uPAR antibody (AF534). Alexa Fluor 488 anti-goat secondary antibody (A11055) was used as the secondary antibody. The quantified mean Alexa 488 fluorescence signal per cell line is presented in the panel to the right. **D and E:** Relative *Plaur* mRNA (uPAR) (**D**) or *Plau* mRNA (uPA) (**E**) expression levels as analysed using RT-qPCR. All expression levels were normalized to the expression of the reference genes *Trfc* and β-*actin*. Error bars represent the standard error of mean (+SEM) and N = 3. One-way ANOVA; *p<0.05. **F:** Plasminogen-gelatin (upper panel) and gelatin (lower panel) zymography analysis of conditioned medium of cells cultured for 24 hours in SFM. HMW-uPA and mPLM (mouse plasmin) were loaded as positive controls. The images were cropped to size. **G:** Relative *Plasminogen* mRNA (Plg) expression levels as analysed using RT-qPCR. Error bars represent standard error of mean (+SEM) and N = 3.

uPAR expression is often increased in OSCCs at the invasive front [40], suggesting that it may have a role in invasion and metastasis. To better understand the role of uPAR in OSCC progression, cells expressing high and low levels of uPAR were generated. The murine OSCC cell line AT84 [41] was selected for this study, as it expresses low endogenous levels of uPAR in culture (figure 1a), and allowed the use of a syngeneic mouse model with immunocompetent mice. Single cell clones expressing high levels of uPAR were generated, and two clones were chosen for further study (uPAR1 expressing very high levels of uPAR, and uPAR2 expressing moderate levels of uPAR, figure 1a). Two single cell clones containing only the empty vector were selected as controls (EV1 and EV2), expressing low endogenous levels of uPAR as shown by Western blotting (figure 1a). Recombinant soluble His-tagged uPAR (rmuPAR) was loaded as a positive control, which (due to being produced in insect cells) is less glycosylated, and therefore has a lower MW than uPAR expressed by the AT84 cells (figure 1a). Cell surface localization of uPAR was verified by Western blotting of membrane fractions (figure 1b), and with flow cytometry on non-permeabilized cells (figure 1c). *Plaur* mRNA levels (figure 1d) reflected the uPAR protein expression levels (figure 1a), and all clones expressed *Plau* mRNA (figure 1e), as analysed by RT-qPCR. Bands with similar size to recombinant active high molecular weight (HMW)-uPA were detected when the conditioned medium was analysed by gelatin-plasminogen zymography, and not by gelatin zymography, indicating that the clones express similar levels of uPA (figure 1f - full gel images can be viewed in figure S1). *Plasminogen* mRNA levels varied among the clones, with EV1 displaying the highest expression, and EV2 the lowest (figure 1g).

### Induction of endogenous uPAR expression *in vivo*

**Figure 2:**
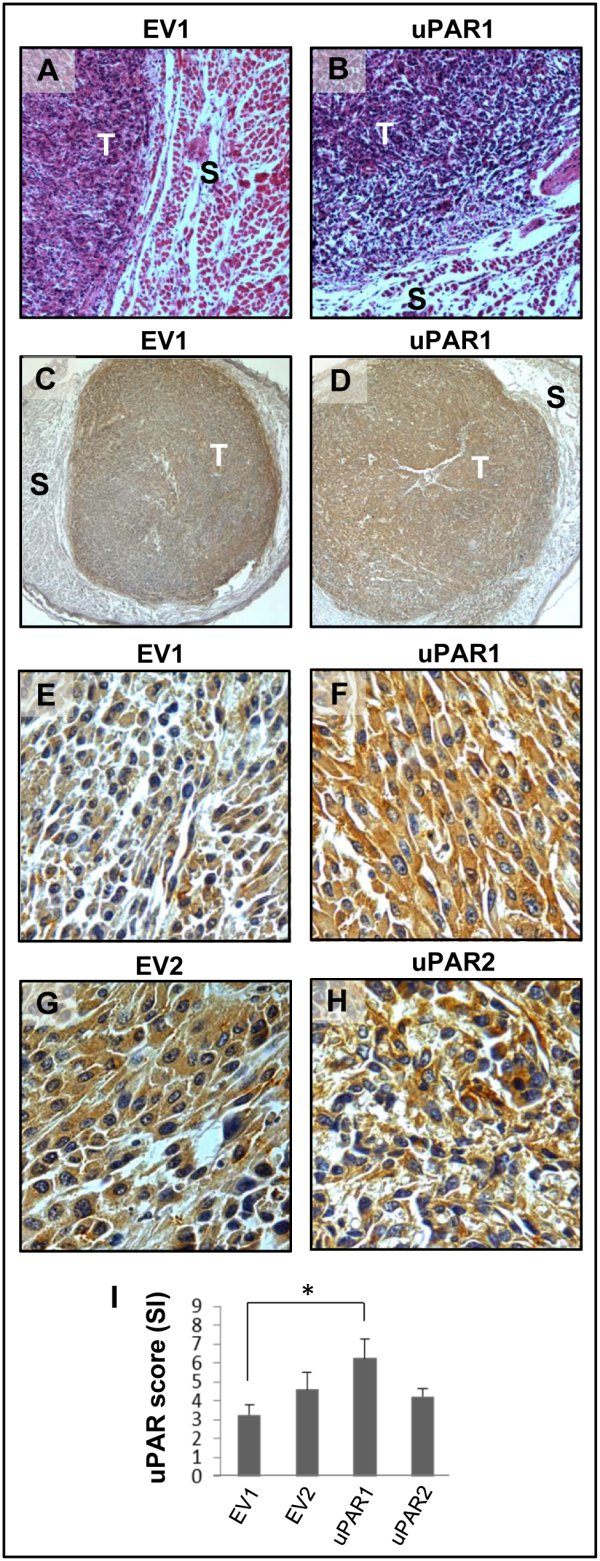
Tumour microenvironment induced uPAR protein expression in tongue tumours. Tumour growth pattern and uPAR protein levels in tongue tumours generated from the EV1, EV2, uPAR1 and uPAR2 cells. **A–B:** Representative images depicting the tumour growth pattern at the tumour-stroma interface in hematoxylin/eosin stained EV1 (**A**) and uPAR1 (**B**) tumours. Images were recorded at 10x magnification. **C–D:** Representative images depicting the IHC uPAR staining of the EV1 (**C**) or uPAR1 tumours (**D**). Images were recorded at 4x magnification. **E–H:** The images show high power magnification (20x magnifications) of the EV1 (**E**), uPAR1 (**F**), EV2 (**G**) and uPAR2 (**H**) tumours IHC stained for uPAR protein. Positive uPAR staining is seen as brown colour, and counterstaining was done with haematoxylin. **I:** The average staining index (SI) of the uPAR staining in the tumours. Maximum obtainable score is 9. The error bars shows the +SEM. N = number of tumours; EV1, N = 8/10; EV2, N = 5/10; uPAR1, N = 4/10; uPAR2 N = 9/10. One-way ANOVA; **p<0.01, *p<0.05. T = Tumours, S = Stroma.

In order to analyse the effects of various levels of uPAR on tumour invasiveness and metastasis, cells expressing either high- (uPAR1 and uPAR2) or low endogenous- (EV1 and EV2) levels of uPAR were injected into the tongue of immunocompetent mice. Tumours were harvested already after 14 days due to rapid tumour growth, although not all mice developed tumours. None of the tumours displayed infiltrative growth and were rounded with clear and defined borders (figure 2a–b). No metastases were detected in lymph nodes, livers or lungs, showing that neither of these clones displayed aggressive behaviour *in vivo* within the limit of 14 days of tumour growth.

Tumours were ZBF-fixed and tissue sections were IHC stained and analysed for the presence of uPAR (figure 2c–h). uPAR staining was mostly seen in the tumour cells both at the cell membrane and in the cytoplasm. To verify the specificity of the uPAR antibody, control experiments were performed where the anti-uPAR antibody was pre-absorbed with recombinant uPAR. IHC staining using the pre-absorbed uPAR-antibody demonstrated that the staining was not due to unspecific binding of the antibody (file S1 and figure S2). Tongue tumours of uPAR1 cells, which in culture express high levels of uPAR, had an average staining index (SI) of 6.25 (out of max 9) and were more positive than tumours of uPAR2 cells (SI = 4.22) (figure 2i) that in culture expressed moderate levels of uPAR. Surprisingly, tumours generated from the EV-cells also displayed a moderately strong staining for uPAR. The EV1 (figure 2e) and EV2 (figure 2g) tongue tumours had an average SI of 3.25 and 4.60, respectively, and were therefore considered to have moderate expression of uPAR similar to uPAR2 (figure 2i). The staining was most prominent in the periphery of the EV-tumours (figure 2c), suggesting that the tumour microenvironment is involved in the up-regulation of uPAR expression.

A similar induction of uPAR expression was observed in the EV-cells when the cells were injected subcutaneously (figure S3), indicating that different types of tumour microenvironments can induce endogenous uPAR expression. The skin tumours, like the tongue tumours, also had clear and defined borders, displayed no infiltrative growth (see figure S3a–b) or metastases. The skin tumours generally displayed weaker uPAR staining in comparison to the tongue tumours with an average SI of 2.44 and 3.90 for the EV1 and EV2 tumours respectively (see figure S3i). This was similar to uPAR2 with an SI of 4.00. The uPAR1 tumours showed the highest SI of 6.38 (see figure S3i).

### Knock-down of uPAR expression in AT84 cells

**Figure 3:**
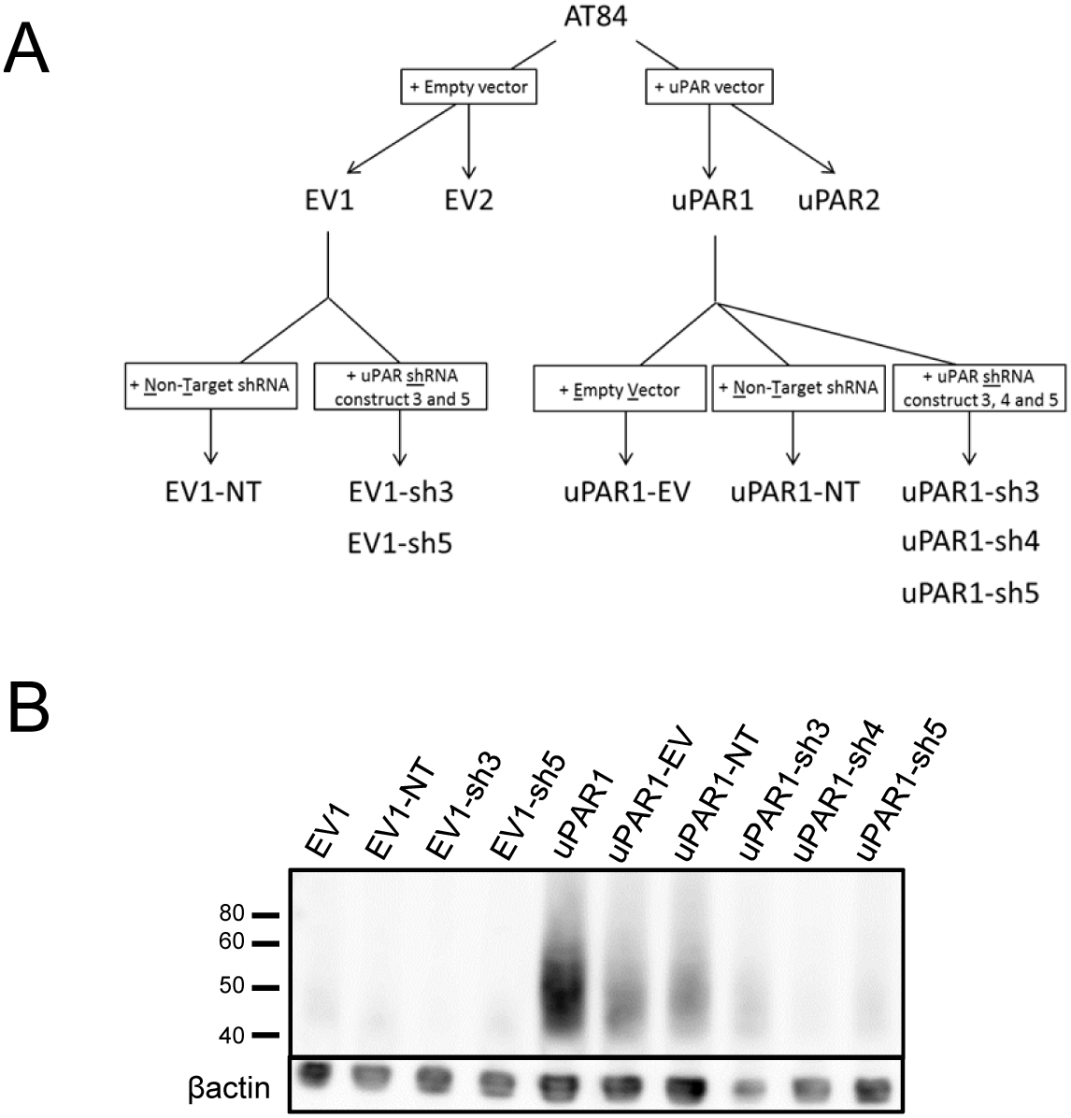
Knock-down of uPAR expression in AT84 cells. shRNA knock-down of *Plaur* in AT84 cells. **A:** Flow chart showing the generation of the single cell clones. **B:** Western blot analysis of whole cell lysates from the single cell clones stably transfected with either shRNA-constructs (shRNA 3 = sh3, shRNA 4 = sh4 or shRNA 5 = sh5) targeting *Plaur* or constructs containing non-target shRNA (NT) or the empty vector (EV). uPAR was detected using a polyclonal anti-murine uPAR antibody (AF534). Re-probing for β-actin was used as a loading control. Images were cropped, as no additional bands were detected in the blot.

Due to the endogenous up-regulation of uPAR *in vivo*, shRNA was transfected into the cells to knock-down and keep uPAR levels low. Five different shRNA constructs were tested by transient transfection of uPAR1 (see figure S4a) cells. New single cell clones were generated based on the EV1 or uPAR1 cells as shown in the flow chart (figure 3a). As controls, EV1 and uPAR1 cells were transfected with either empty vector (EV) or non-target shRNA (NT). The selected EV1-NT, EV1-sh3 and EV1-sh5 single cell clones all displayed low or undetectable levels of uPAR on Western blots (figure 3b). The selected control cells uPAR1-EV and uPAR1-NT displayed some reduction in uPAR levels when compared to the uPAR1 cells, but a much greater reduction was obtained in the uPAR1-sh3, uPAR1-sh4 and uPAR1-sh5 knock-down cells (figure 3b). A mixed population of shRNA bulk transfected cells (uPAR-sh-B) was also generated, though the efficiency of the uPAR knock-down was less than that obtained with single cell cloning (see figure S4b and file S2). Thus, the single cell clones were therefore chosen for the subsequent experiments.

### *In vivo* tongue tumours of uPAR knock-down cells

**Figure 4:**
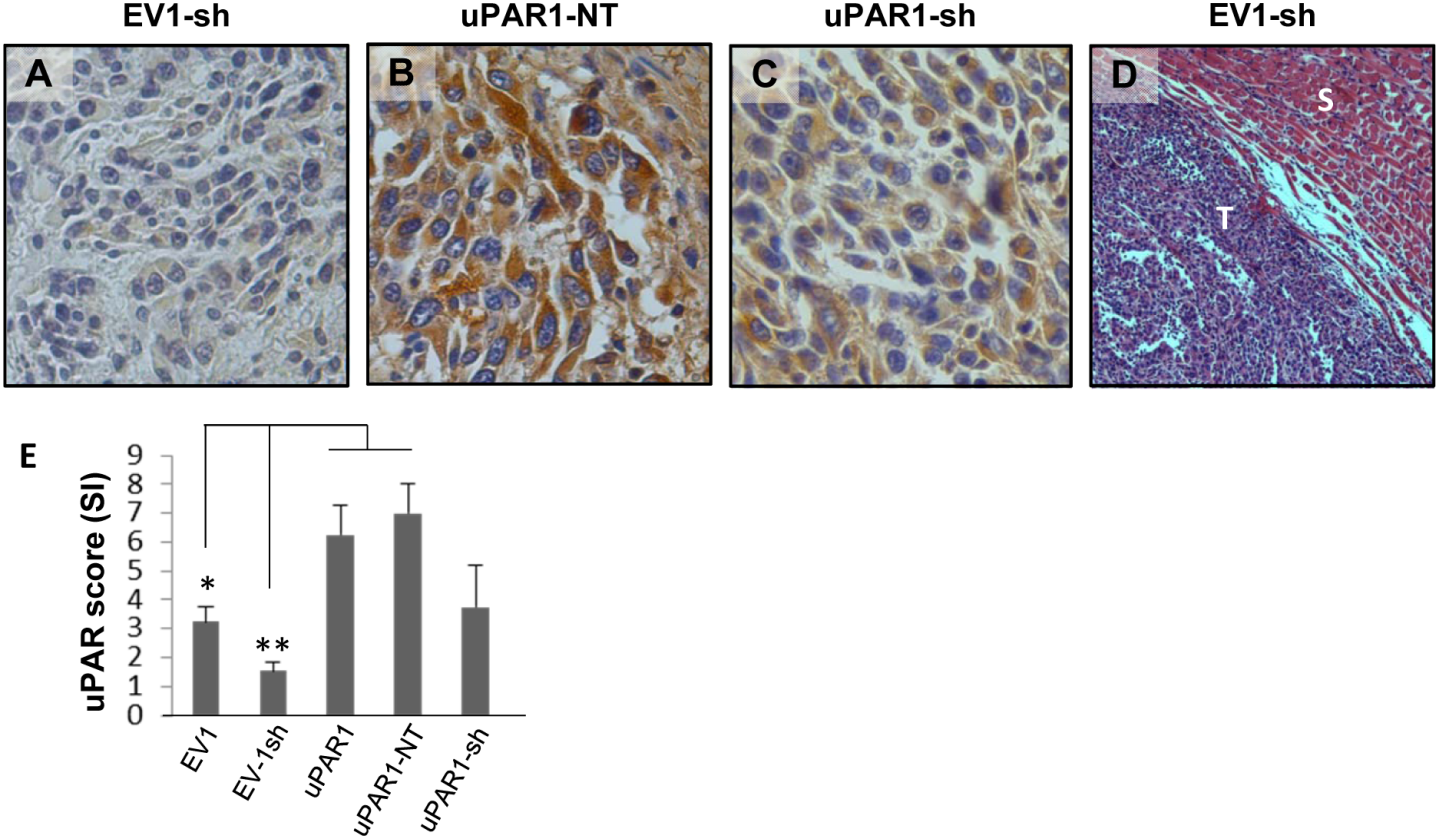
*In vivo* tongue tumours of EV1 and uPAR1 knock-down cells. IHC uPAR staining and growth pattern of tongue tumours generated from the EV1 and uPAR1 cells containing either shRNA targeting uPAR (EV1-sh and uPAR1-sh), or non-targeting shRNA (uPAR1-NT). **A–C:** IHC uPAR staining of EV1-sh (**A**), uPAR1-NT (**B**) and uPAR1-sh (**C**) tumours, respectively. Images were recorded at 20x magnifications. **D:** Representative image depicting the tumour growth pattern at the tumour-stroma interface in hematoxylin/eosin stained EV1-sh. **E:** The average SI of the uPAR staining in the tumours, with the maximum obtainable score of 9. The error bars shows the +SEM. N = number of tumours; EV1, N = 8/10; EV1-sh, N = 11/16; uPAR1, N = 4/10; uPAR1-NT, N = 3/8; uPAR1-sh, N = 4/16. One-way ANOVA; **p<0.01, *p<0.05. T = Tumour, S = Stroma.

Tongue tumours were generated with the new clones in order to analyse the effects on invasiveness and uPAR expression in the presence of *Plaur*-targeting shRNA. For the analysis of the tumours, the EV1-sh3 and EV1-sh5 tumours were grouped together as EV1-sh, and the uPAR1-sh4 and uPAR1-sh5 tumours were grouped together as uPAR1-sh. IHC staining revealed that the uPAR levels were kept low in the EV1-sh tumours (figure 4a). The average SI of the uPAR staining is presented in the graph in figure 4e. The EV1-sh tumours had an average SI of 1.55 and showed considerably lower levels of uPAR than the EV1 tumours (SI = 3.25), and a significantly lower expression than the uPAR1-NT tumours (figure 4b) which had an average SI of 7 (figure 4e). The uPAR1-sh tumours (figure 4c) displayed great variations in uPAR protein expression, resulting in a large standard error of mean (figure 4e). Tumours from the EV1-sh cells (figure 4d), as well as tumours from the control transfected cells (uPAR1-NT) (data not shown), displayed no signs of infiltrative or metastatic growth. Taken together, although shRNA mediated knock-down of uPAR enabled generation of tumours with significantly different levels of uPAR, no difference in tumour invasiveness or metastasis could be detected.

### Leiomyoma stroma is a strong inducer of uPAR expression in invading cells

**Figure 5:**
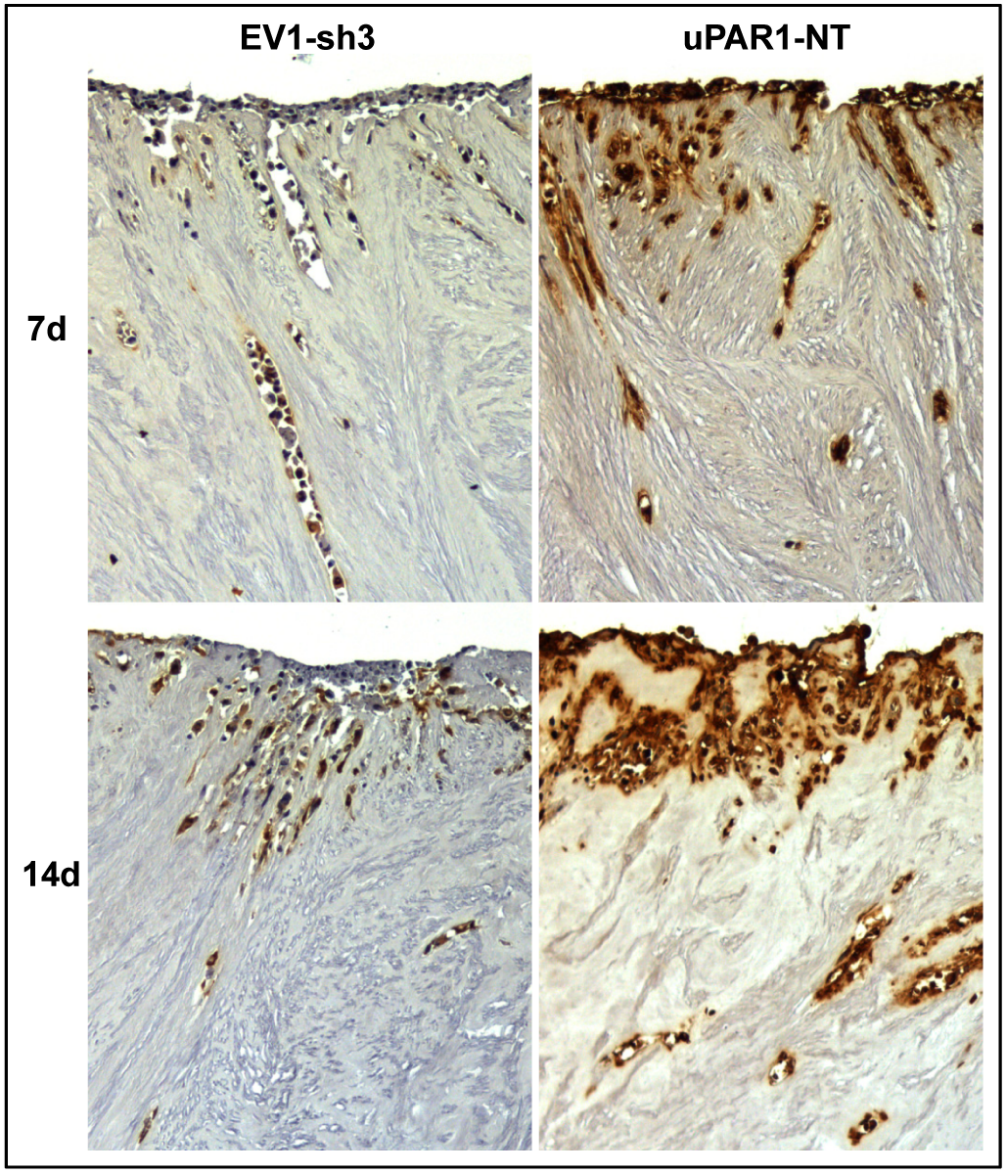
Leiomyoma stroma is a strong inducer of uPAR expression. Representative images of low- (EV1-sh3) and high- (uPAR1-NT) uPAR-expressing cells invading the *ex vivo* leiomyoma tissue. Cells were incubated for 7 and 14 days, as indicated. The tissue was IHC stained for uPAR. Positive uPAR staining is seen as brown colour, counterstained with haematoxylin. Images were recorded at 10x magnification.

In order to more specifically analyse the effects of the tumour microenvironment on uPAR expression and invasive capacity, the leiomyoma invasion model was used [39]. The human neoplastic leiomyoma tissue is rich in collagen I, -III, -IV, and laminins, and this organotypic invasion model has proven to be a good model for local invasion, mimicking the hypoxic tumour environment [42]. Cells expressing either high- or low levels of uPAR were seeded on top of the leiomyoma tissue discs and incubated for 7 or 14 days, whereupon the leiomyoma tissue was ZBF-fixed. Tissue sections were stained with H/E and total invasion was scored (file S3). No differences in invasion that could be directly attributed to the uPAR expression status of the cells were found (figure S5). In order to determine the uPAR expression levels in the cells invading the leiomyoma tissue, they were IHC stained for uPAR (figure 5). Negative controls, with no added cells, gave no uPAR staining (results not shown). The high-uPAR expressing clones (uPAR1-EV and uPAR1-NT) remained uPAR positive throughout the experiment regardless of whether the cells were located on top or invading into the tissue (figure 5, right panels). In contrast, the low uPAR expressing cells (EV1-sh3 and EV1-sh5) located on top of the leiomyoma tissue remained uPAR negative for the duration of the experiment, while invasive cells gradually increased the uPAR protein levels with time despite the presence of shRNA constructs (figure 5, left panels). Thus, invading cells were strongly induced to express uPAR, further implicating the tumour microenvironment in regulation of uPAR expression.

### Soluble factors from the leiomyoma tissue mediate a time dependent induction of uPAR levels

**Figure 6:**
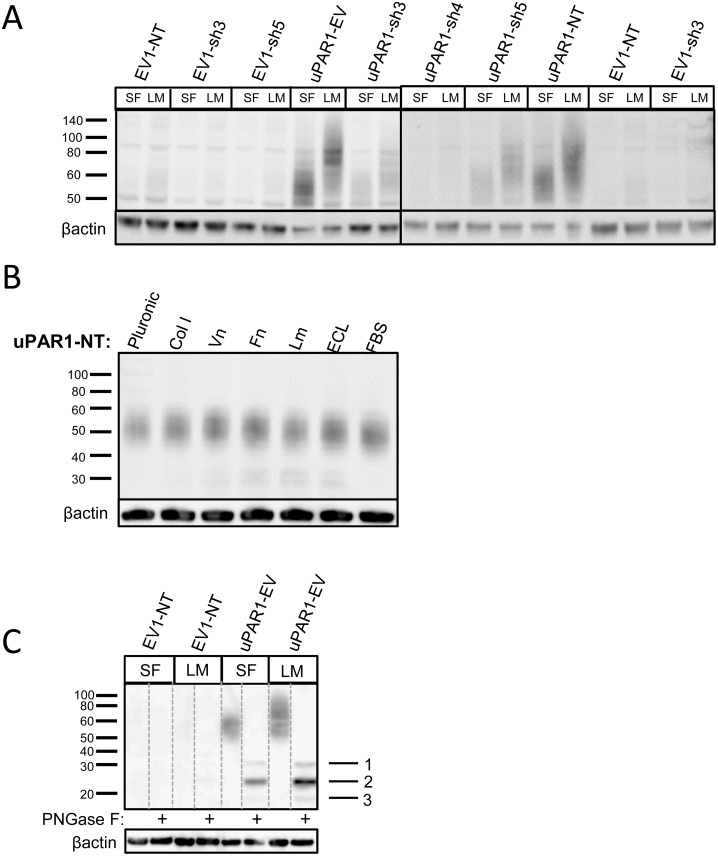
Leiomyoma conditioned medium induced uPAR expression. Analysis of uPAR expression induced by the LCM or purified ECM proteins in cultured uPAR knock-down cells. All Western blots were performed on whole cell lysates, and uPAR was detected using the polyclonal anti-murine uPAR antibody (AF534). **A:** Cells were either cultured in LCM (LM) or serum free medium (SF) for 24 hours. Cells were harvested with sample buffer and re-probing for β-actin was used as a loading control. **B:** uPAR1-NT cells were seeded on different ECM protein substrates, incubated for 24 hours and harvested using RIPA buffer. 7.5 µg of total protein was loaded per lane. Equal loading was verified by re-probing for β-actin. The poloxamer pluronic was used as a no-adhesion control. Col I = Collagen I, Vn = Vitronectin, Fn = Fibronectin, Lm = Laminin, ECL = Entactin, Collagen, Laminin, and FBS = Foetal Bovine Serum. **C:** Cells cultured in LCM (LM) or serum free medium (SF) for 24 hours were harvested using sample buffer and deglycosylated by PNGase F treatment (+) as indicated. Re-probing for β-actin was used as a loading control. The three bands detected by the anti-uPAR antibody are labelled 1, 2, and 3, respectively.

The up-regulated expression of uPAR seen in the EV-cells could be due to either soluble or insoluble factors present in the tumour microenvironment. Many different growth factors, cytokines and chemokines are known to up-regulate uPAR expression in various cell types [43]–[46]. To analyse whether soluble factors from the leiomyoma could explain the increased expression of uPAR, the freeze-dried leiomyoma tissue was rehydrated in serum free medium (SFM) which was subsequently used as conditioned growth medium for the cells in culture. After 24 (figure 6a) or 48 hours (figure S6), the cells were harvested and analysed for uPAR expression by Western blotting. uPAR levels were not notably increased in the uPAR1-EV and uPAR1-NT cells, although the size of the expressed uPAR was strikingly increased (figure 6a). Little uPAR could be detected in the low uPAR expressing clones (EV1-NT, EV1-sh3 and EV1-sh5) after 24 or 48 hours of incubation with leiomyoma conditioned medium (LCM). There was, however, a small induction of uPAR expression in the uPAR1-sh3 and uPAR1-sh5 clones after 24 hours (figure 6a). Incubating the cells in LCM for 48 hours induced a marked increase in uPAR expression levels in all the uPAR1-sh cells (figure S6), indicating that these cells more readily turn on the uPAR expression, as also seen *in vivo* (figure 4).

To further test whether ECM proteins known to be present in the tumour stroma could regulate the expression of uPAR, EV1-NT, EV1-sh3, uPAR1-NT and uPAR1-sh4 cells were seeded on different ECM substrates. Western blot analysis showed that none of the substrates induced detectable levels of uPAR in the EV1-NT, EV1-sh3 and uPAR1-sh4 cells (results not shown), whereas uPAR1-NT cells displayed equal levels of uPAR on all substrates tested (figure 6b).

To investigate whether the size change induced by the LCM was due to increased glycosylation, lysates of cells that had been treated with LCM or SFM were deglycosylated by PNGase F treatment (figure 6c). Western blot analysis using the polyclonal anti-uPAR antibody showed that cells treated with either SFM or LCM expressed three distinct bands after deglycosylation (figure 6c, indicated with the numbers 1, 2 and 3). Band no. 1 corresponds to the size previously reported for full length deglycosylated uPAR (approximately 35 kDa). Band no. 2 corresponds to the previously reported size of deglycosylated uPAR D2+D3 (approximately 25 kDa) [14]. Band no. 3 could possibly correspond to either D1 of uPAR or GPI-anchored D3 of uPAR (approximately 18 kDa) [16], [19]. Hence, the increased size of uPAR after incubation with LCM was either due to increased glycosylation, or possibly due to less cleavage of full-length uPAR. Analysis of the bands by mass spectrometry revealed that uPAR-peptides were present in both band no. 1 as well as in band no. 2 (results not shown). No uPAR-peptides could be detected in the 18 kDa band. To verify that bands no. 1 and 2 represent full-length and cleaved uPAR, respectively, cells were incubated with the uPA inhibitor BC11 hydrobromide. uPA is reported to cleave the linker region between D1 and D2 of uPAR [14], hence producing uPAR D2+D3. Cells expressing high levels of uPAR were incubated in the presence of BC11 hydrobromide for 72 hours, harvested and analysed by Western blot using the anti-uPAR antibody (file S4 and figure S7). By inhibiting uPA, band no. 1 became stronger than band no. 2, indicating that the two bands indeed represent full-length and cleaved uPAR, respectively.

Taken together, soluble factors isolated from the leiomyoma tissue induced an increase in uPAR levels in a time dependent manner. In addition, the LCM induced an increase in the size of uPAR probably due to increased glycosylation, and possibly also altered the cleavage of uPAR.

### Increased activity of gelatinolytic enzymes at the invasive front

**Figure 7:**
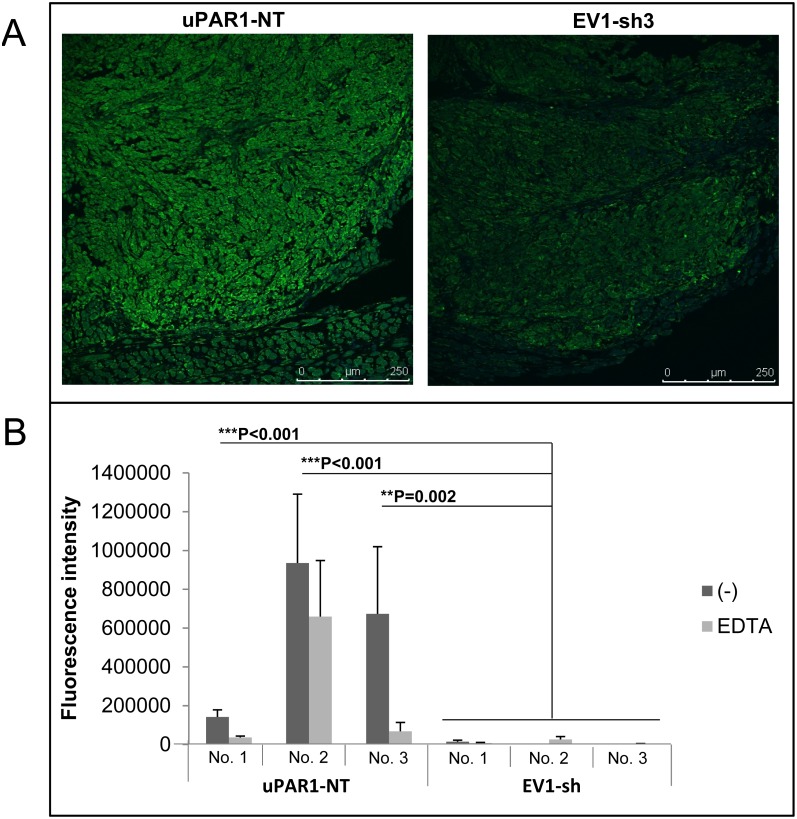
Gelatinolytic activity is enhanced in tumours expressing high levels of uPAR. ZBF-fixed tongue tumours were sectioned and analysed for the presence of gelatinolytic activity using DQ-gelatin *in situ* zymography. Gelatinolytic activity is seen as green fluorescence. **A:** Representative confocal images of tongue tumours generated from the uPAR1-NT cells (left panel) and EV1-sh3 cells (right panel). **B:** Quantification of fluorescence intensity (analysed using Volocity as described in materials and methods) for a minimum of 5 images per tumour, presented as mean values. Three individual uPAR1-NT tumours (No.1–No.3) and three EV1-sh tumours (No.1–No.3) were analysed. Error bars shows the standard deviation (+SD) between the five images analysed. Dark grey bars represents gelatinolytic activity in the tumour sections, light grey bars represents gelatinolytic activity in tumour sections treated with the metalloproteinase inhibitor EDTA. Mann-Whitney rank sum test; ***p<0.001, **p<0.01, *p<0.05.

Breakdown of surrounding stroma is a key step in the process of tumour invasion and metastasis. Plasmin, in addition to gelatinases such as MMP-2 and MMP-9, are known to be potent gelatin degrading enzymes. Furthermore, gelatinolytic enzymes have many non-ECM substrates which might have a role in cancer progression. To assess whether the regulation of uPAR expression affects the cell’s ability to activate gelatinolytic enzymes, the tumours were analysed by *in situ* zymography. Sections of tongue tumours generated from EV1-sh3, EV1-sh5 and uPAR1-NT were incubated with dye quenched (DQ)-gelatin and analysed by confocal microscopy. The results showed a marked increase in active gelatinolytic enzymes in the tumours expressing high levels of uPAR (figure 7a). Auto-fluorescence was undetectable as assessed by incubating the sections at −20°C for 2 hours immediately after the substrate was added (results not shown). The gelatinolytic activity was quantified using Volocity software (figure 7b), and a statistically significant difference in gelatinolytic activity was found between the EV1-sh and the uPAR1-NT tumours. In order to distinguish between gelatinolytic activity of metalloproteases and activity from other gelatin degrading enzymes such as plasmin, the metalloprotease-inhibitor EDTA was added. EDTA reduced the activity to some extent, though with varying degrees from tumour to tumour (figure 7b). Thus the enzymes contributing to the gelatinolytic activity are a mixture of metalloproteases and non-metalloproteases. Tumours generated from uPAR1 and EV1 cells (figure 2) were also analysed by *in situ* zymography (figure S8). Also in these tumours the gelatinolytic activity was significantly increased in the uPAR1 tumours compared to the EV1 tumours though the difference was not as clear, possibly due to the up-regulation of uPAR in the EV1 cells *in vivo* (see figure 2a).

**Figure 8:**
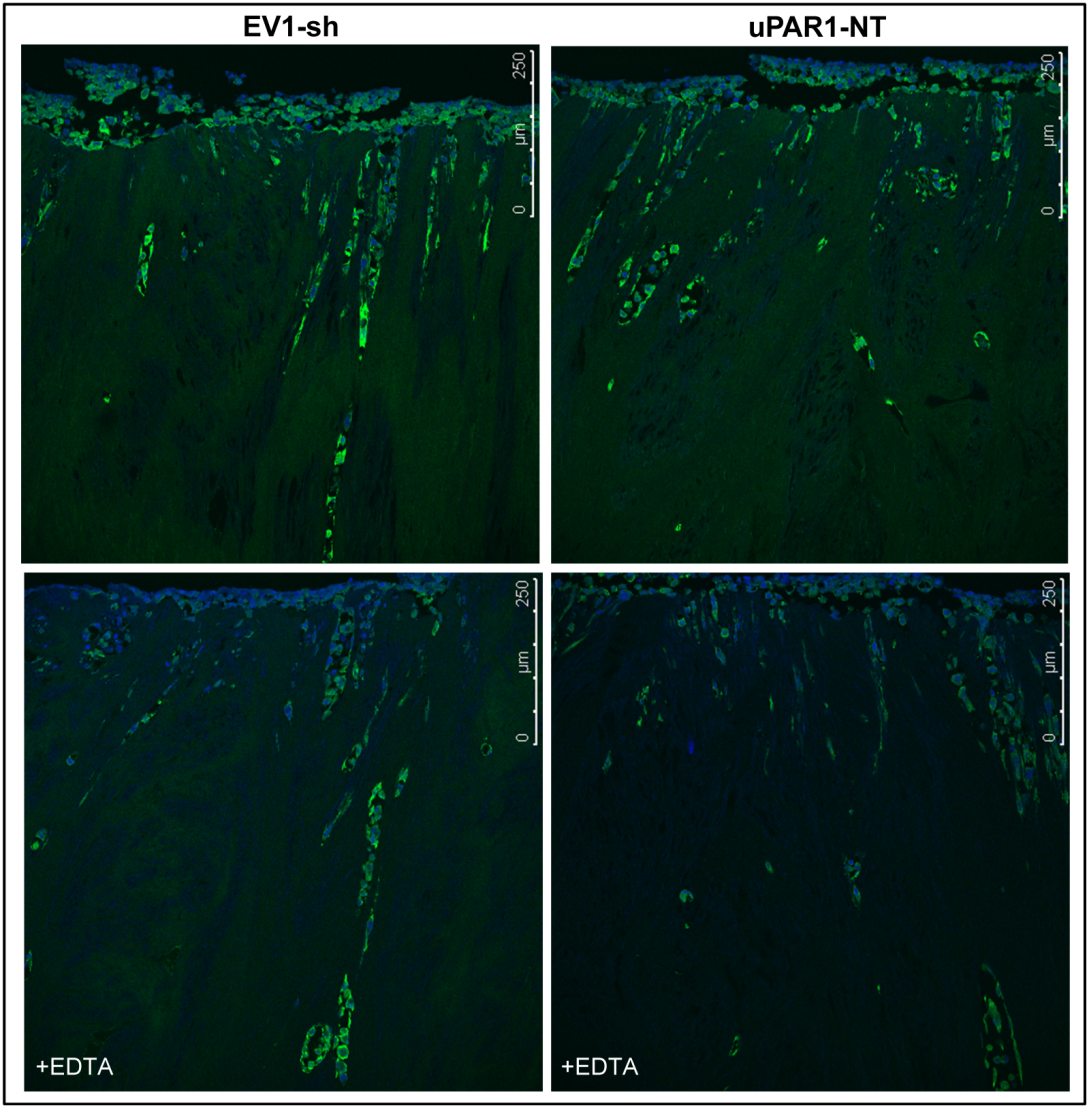
Gelatinolytic activity is enhanced in cells invading leiomyoma tissue. ZBF-fixed leiomyoma tissue was sectioned and analysed for the presence of gelatinolytic activity using DQ-gelatin *in situ* zymography. Gelatinolytic activity is seen as green fluorescence, nuclei are stained blue with DAPI. Representative confocal images of cells expressing either low- (EV1-sh3, left panels) or high (uPAR1-NT, right panels) levels of uPAR invading the *ex vivo* leiomyoma tissue. The upper panels show gelatinolytic activity in the tissue, while the lower panels show tissue sections treated with the metalloproteinase inhibitor EDTA.

In addition, gelatinolytic activity was assessed in high- and low uPAR-expressing cells invading the leiomyoma tissue (figure 8). There was no significant difference between the clones, but the gelatinolytic activity was generally stronger in the invading cells compared to the non-invading cells. This is in accordance with the finding that all invading cells showed elevated uPAR expression regardless of the uPAR level of the clones *in vitro* (see figure 5). When EDTA was added, only the gelatinolytic activity of the non-invading cells, where uPAR levels were low, was reduced. Meanwhile, there was no inhibition seen in the gelatinolytic activity of the invading cells which had high uPAR expression. Taken together, tumour cells with increased uPAR levels also displayed increased ability to activate gelatinolytic enzymes.

## Discussion

uPAR and uPA have both been linked to poor prognosis for several cancer types, where they are thought to play a role in invasion and metastasis [5]–[7]. In light of this, the main focus of the current study was to elucidate the role of uPAR expression in OSCC. To this end, uPAR was first overexpressed in the murine OSCC cell line AT84 (figure 1), and cells were analysed *in vitro* in cell culture, *in vivo* as tongue and skin tumours (figure 2 and figure S3), and *ex vivo* when invading leiomyoma tissue (figure 5 and 8). The main finding was that the uPAR levels of the tumour cells did not affect the invasiveness, and that uPAR expression was readily up-regulated by the tumour microenvironment both *in vivo* in the tongue and *ex vivo* in the leiomyoma tissue. Furthermore, we observed that cells with high uPAR expression displayed increased gelatinolytic activity (figure 7 and 8).

### Microenvironment induced uPAR expression

Analysis of the tongue- and skin tumours generated from cells expressing low endogenous levels of uPAR, revealed that these cells had up-regulated uPAR protein levels *in vivo* (EV1 and EV2 in figure 2). The IHC staining for uPAR was most prominent in the periphery of these tumours (figure 2c), where the cells were in contact with stromal cells including several types of immune cells. Furthermore, cells invading the tissue of the leiomyoma also showed enhanced uPAR expression (figure 5). These tumour microenvironments are potential storage depots of cytokines, chemokines and different growth factors such as transforming growth factor β (TGFβ), epidermal growth factor (EGF), basic fibroblast growth factor (bFGF) and vascular endothelial growth factor (VEGF) [47]–[49], many of which have been shown to up-regulate the expression of uPAR [43]–[46], [50], [51]. This could explain why the expression is stronger along the tumour-stroma interface, and in leiomyoma invading cells.

### Specificity of the anti-uPAR antibody

The uPAR staining seen in the tumour cells was mostly located intracellularly (figure 2), which prompted analysis of the specificity of the anti-uPAR antibody used (file S1, figure S2). As the results showed that the antibody is highly specific for uPAR, the apparent lack of expected membrane staining could be explained by several factors, where uPAR cleavage either partially (inter-domain cleavage) or by complete shedding (cleavage of the GPI-anchor) from the cell surface is one option [17]. Both phospholipase C and D can cleave the GPI-anchor of uPAR [24], [26], giving rise to soluble uPAR (suPAR). The proportion of cell surface located uPAR is also regulated by the rate of endocytosis. Both low-density lipoprotein (LDL) receptor-related protein 1 (LRP1) and Endo180/uPAR-associated protein (uPARAP) are involved in turnover of uPAR. Although most uPAR is recycled back to the cell surface, very active endocytosis could eventually deplete the fraction of uPAR at the plasma membrane [52], [53]. Thus, the activity of both LRP1 and Endo180/uPARAP could very well influence the cell surface levels of uPAR.

### Soluble factors induce altered posttranslational modifications of uPAR

To further investigate whether the soluble factors, such as growth factors in the leiomyoma tissue were involved in the regulation of uPAR expression, leiomyoma conditioned medium was used to stimulate the cells (figure 6a). Soluble factors present in the conditioned medium were able to override the shRNA in the uPAR1 knock-down cells, and gradually increase the expression of uPAR with time. Further experiments are needed to identify the molecular mechanisms for this effect. Interestingly the conditioned medium induced a marked increase in the molecular weight of uPAR when compared to control cells treated with SFM. With five functional, and two potential, N-linked glycosylation sites reported for murine uPAR [54], an alteration in the glycosylation pattern could explain the increase in size. This is interesting as modifications of the glycosylation pattern can enhance uPAR’s ability to bind and activate uPA [55], [56]. In addition, more highly glycosylated variants of uPAR have been observed in malignant thyroid tumour cells when compared to normal thyroid cells [57]. Different cell types also express different glycosylated variants of uPAR, and the glycosylation pattern can be altered in response to different stimuli, such as PKC activation as shown by PMA stimulation [58]. Ragno et al. [59] also reported that the increased glycosylation of uPAR seen in the thyroid tumour cells rendered uPAR less susceptible to cleavage by uPA, plasmin and chymotrypsin. It has also been reported that cleavage-resistant uPAR is less efficiently cleared from the cell surface [60]. These are all events that could potentially increase the pericellular proteolysis of the tumour cells. Deglycosylation of the LCM treated and SFM treated cells revealed three distinct bands (figure 6c, band no. 1–3), indicating that uPAR was cleaved. Cells treated with LCM displayed more of uPAR D2+D3 (band no. 2), but also to some extent showed more of full-length uPAR (band no. 1). Thus, our finding that a large proportion of the expressed uPAR is cleaved is not consistent with the hypothesis that increased glycosylation protects uPAR against proteolytic cleavage. However, this might be cell type specific since N-linked glycans are known to be very heterogeneous in structure and that many proteins exist in various glycoforms in different diseases [61]. Thus, it is therefore still uncertain whether the shift in the molecular weight of uPAR that we observed was due to a change in glycosylation, or whether it is due to increased levels of full length uPAR.

### High uPAR levels increases the activity of gelatinolytic enzymes

A stromal-induced alteration in expression levels, glycosylation and/or cleavage of uPAR could potentially affect the pericellular proteolysis of tumour cells. Cleavage between D1 and D2 of uPAR renders it unable to bind pro-uPA, and may therefore represent a natural regulatory mechanism to avoid overactive proteolysis [7]. It might also reflect a highly active PA system, as uPA and plasmin can both cleave uPAR [14]. Whether the expressed uPAR in the tumours or the cells invading the leiomyoma tissue display altered glycosylation and/or cleavage has not been investigated. However, the results obtained using the LCM suggests that such modifications are plausible. When tongue tumours were examined for gelatinolytic activity, tumours expressing high levels of uPAR displayed a substantial ability to activate gelatinolytic enzymes compared to tumours with low uPAR levels (figure 7). Similar results were obtained when the cells invading the leiomyoma tissue were examined (figure 8). Cells invading deep into the tissue had up-regulated uPAR levels and displayed an increased ability to degrade gelatin, hence activate gelatinolytic enzymes. EDTA-treatment of leiomyoma tissue sections indicated that the activity seen in the invading cells mainly originates from non-metalloproteinases such as uPA and plasmin, underscoring a role for uPAR and the stroma in the regulation of gelatinolytic activity. On the other hand other enzymes such as trypsin and cathepsins could also cause the gelatin degradation seen in the tumours and the invading cells, and further investigations are needed in order to reveal the identity of the proteolytic enzymes involved. Tumours expressing either high- or low levels of uPAR displayed similar growth patterns with few signs of aggressive behaviour. This indicates that the expression of uPAR and subsequent activation of gelatinolytic enzymes is not sufficient to induce infiltrative growth and metastatic behaviour of the AT84 cells in this *in vivo* tumour model. Several in vitro studies have suggested that suPAR could function as an inhibitor for tumour progression, scavenging the active uPA [62], [63]. Whether this is the case in our tumour model is an interesting possibility. Thus, further studies on the role of uPAR cleavage and glycosylation in relation to tumour invasion and metastasis formation are warranted.

## Conclusions

Taken together, we have observed that the tumour microenvironment is involved in the induction of uPAR expression. Furthermore the increased expression of uPAR, either by overexpression or by natural up-regulation, increased the activity of gelatinolytic enzymes in these cells, however this did not affect the tumour invasiveness in our mouse model. Further studies on the observed effects of the tumour microenvironment on expression and post-translational modifications of uPAR are warranted. Unravelling the biological significance of posttranslational modifications of uPAR, as well as the mechanisms regulating them, might provide answers to why uPAR is often associated with poor prognosis in many types of cancers.

## Methods

### Ethical statement

The experimental protocol was approved by the competent local authority reporting to the Norwegian National Animal Research Authority, project licence no. FOTS 2598 and 4020/date of approval 27.04.2010 and 31.01.2012, respectively. All animal procedures were carried out in accordance with the Norwegian Regulations on Animal Experimentation (REG 1996-01-15 no. 23) and in agreement with European Convention for the Protection of Vertebrate Animals used for Experimental and Other Scientific Purposes (Convention No. 123 Issued by the Council of Europe) [64]. Experimental applications included experimental set-up, rationale for the experiment, and efforts made to refine, replace and reduce the animal experiments. The work is reported according to the ARRIVE guidelines [65]. Use of patient material (leiomyoma tissue) was approved by the Northern Ostrobothnia Hospital District Ethics Committee (statement #8/2006 and amendment 19/10/2006), with written informed consent from the donors [66].

### Cell culture

The mouse tongue SCC cell line AT84, originally isolated from a C3H mouse [41], was kindly provided by Professor Shillitoe, Upstate Medical University, Syracuse, NY [67]. AT84 has previously been reported as invasive and metastatic when injected into the floor of the mouth through an extra-oral route [67]. Cells were cultured at 37°C, 5% CO_2_ in a humid environment in NaHCO_3_-buffered RPMI-1640 (R8758, Sigma Aldrich, St. Louis, USA) supplemented with 10% FBS (F7524, Sigma Aldrich, St. Louis, USA). For AT84 cells overexpressing uPAR, the culture medium was supplemented with 5 µg/ml puromycin dihydrochloride (P9620, Sigma Aldrich, St. Louis, USA). uPAR shRNA knock-down cells were cultured in RPMI-1640 supplemented with 10% FBS, 5 µg/ml puromycin dihydrochloride (Sigma Aldrich, St. Louis, USA) and 300 µg/ml G418 (G8168, Sigma Aldrich, St. Louis, USA). Cells were also routinely checked for mycoplasma infections.

### Cloning and overexpression of mouse *Plaur*

The mouse gene for uPAR, *Plaur*, was cloned using the Gateway cloning system (Invitrogen, CA, USA). RNA from mouse J774 macrophage cells was used as template and *Att*B-kozac-uPARmouse Fw primers (GGGGACAAGTTTGTACAAAAAAGCAGGCTTCGCCACCATGGGTCACCCGCCGCTGCTGCCG) and *Att*B-uPAR-mouse Rev primers (GGGGACCACTTTGTACAAGAAAGCTGGGTTTAGGTCCAGAGGAGAGTGCCTCCCCA) were used to make the Gateway *att*B-PCR product. Entry clones (pENTR/kozac/uPARmouse) were created by cloning the *att*B-PCR product into the pDONR-221 vector by BP clonase. The destination vector pcDNA5/FRT/TO (Invitrogen, Paisley, UK) was modified by replacing the FRT-site and the neomycin cassette with reading frame A (RfA) (Invitrogen, Paisley, UK) and the puromycin resistance gene controlled by the PGK promoter from the pLKO.1 vector (Thermo Scientific, Wilmington, USA), and termed pDest/TO/PGK-puro. The uPAR PCR-product was transferred to the destination vector by LR clonase and named pDest/TO/PGK-puro/uPAR. The pDest/TO/PGK-puro/uPAR, and pDest/TO/PGK-puro as control, were linearized by ScaI restriction enzyme digestion and transfected into AT84 cells using Lipofectamine LTX & Plus reagent (Invitrogen, Carlsbad, USA). Successfully transfected cells were selected in culture medium supplemented with 5 µg/ml puromycin dihydrochloride (Sigma Aldrich, St. Louis, USA), and single cell clones were expanded for further work. Two clones expressing high levels of uPAR were selected and named uPAR1 and uPAR2, and two clones containing only the empty vector and hence expressing low levels of uPAR were selected and named EV1 and EV2.

### shRNA knock down of mouse *Plaur*

Constitutive knock-down of *Plaur* was achieved using MISSION shRNA Plasmid DNA (NM_011113, Sigma Aldrich, St. Louis, USA). Five different shRNA constructs TRCN0000088818 (construct 1), TRCN0000294900 (construct 2), TRCN0000294902 (construct 3), TRCN0000362694 (construct 4) and TRCN0000362760 (construct 5) under the control of the U6 promoter of the pLKO.1-neo vector were purchased from Sigma Aldrich (St. Louis, MO, USA). An empty pLKO.1-neo vector (EV) and a pLKO.1 vector containing non-target shRNA (NT) were used as controls. The constructs were stably transfected into uPAR1 and EV1 single cell clones using Lipofectamine 2000 (Cat# 11668-019, Invitrogen, Carlsbad, USA). Successfully transfected cells were selected in culture media supplemented with 5 µg/ml puromycin dihydrochloride and 1 mg/ml G418, and single cell clones were expanded for further work. The selected clones were given names as listed in the flow chart in figure 3b.

### Antibodies

Antigen affinity-purified polyclonal goat anti-mouse uPAR antibody (AF534) was from R&D Systems (Minneapolis, MN, USA) and used at 1∶100 in flow cytometer analysis, 1∶200 in immunohistochemistry (IHC) for 1 hour at room temperature, and 1∶1000 in Western blotting. For flow cytometery, the Alexa Fluor 488 donkey anti-goat antibody (A11055) from Invitrogen (Carlsbad, USA) was used at 1∶500. For Western blotting, HRP-conjugated anti-goat/sheep (A9452) was used at 1∶100.000, and HRP-conjugated anti-β-actin (A3854) at 1∶25000 (Sigma Aldrich, St. Louis, USA).

### Western blotting

Cells were detached using trypsin (0.25% in PBS with 0.05% Na_2_EDTA), counted and seeded according to the specific assay. Untreated cells were seeded in serum-containing medium and incubated for 24 hours. For the ECM protein assay, plates were coated for 1 hour at 37°C with either Pluronic F108NF Prill Poloxamer 338 (BASF Corporation, Florham Park, NJ, USA) and fibronectin which was a kind gift from Professor Staffan Johansson, Uppsala University Sweden, collagen I (#C3867-1VL, Sigma Aldrich, St. Louis, MO, USA), vitronectin was purified from human blood as previously described [68], laminin (Lm) (#08–125, Upstate, Lake Placid, NY, USA), entactin, collagen and laminin (ECL) cell attachment matrix (#08–110, Upstate, Lake Placid, NY, USA), 10% FBS in RPMI-1640 medium, 1∶5 dilution of Matrigel (#BS6234) and growth factor reduced (GFR) Matrigel (#BS354230) (BD Biosciences, Bedford, MA, USA). Cells were seeded in serum-free RPMI-1640 medium (SFM) and incubated for 24 hours. For the urokinase inhibitor experiment, cells were seeded in serum-containing medium and incubated for 24 hours. Media was replaced with fresh serum-containing medium with either 10 µM, 20 µM or 30 µM BC11 hydrobromide (#4372, Trocris Bioscience, Ellisville, MO, USA), or no added inhibitor as a control. Cells were incubated for 72 hours and fresh medium containing inhibitor was added every 24 hours. For the LCM-experiment, cells were seeded in serum containing medium. After 24 hours the medium was exchanged either for SFM or conditioned medium (see “organotypic invasion model”). Cell lysates were prepared by removing the culture medium and adding either RIPA buffer (25 mM Tris-HCl pH 7.6, 150 mM NaCl, 1% Triton, 1% sodium dexycholate, 0.1% SDS) with added 1x SIGMAFAST Protease inhibitor (S8830, Sigma Aldrich, St.Louis, MO, USA), or samples buffer (0.05 M Tris-HCl pH 6.8, 2% SDS, 10% glycerol, 0.1% bromphenol blue) and cells were harvested by scraping. Cells harvested with RIPA buffer were added 1x samples buffer. All cell lysates were sonicated and boiled before the samples were loaded onto NuPAGE Novex 4%–12% Bis-Tris gels (Invitrogen, Eugene, USA), and subjected to reducing (PNGase F treated cells) or non-reducing SDS-PAGE. Recombinant mouse soluble His-tagged uPAR was loaded as a positive control in some experiments (CSI20008, Cell Sciences, Canton, MA, USA). Proteins were blotted onto PVDF membranes (Millipore Corp., Bedford, MA, USA). Blocking was done with 5% non-fat dry milk in Tris-buffered saline (150 mM NaCl, 20 mM Tris, pH 7.4) supplemented with 0.1% Tween 20. Membranes were incubated with primary antibody recognizing mouse uPAR (AF534). For some experiments the total protein concentration was measured using the Direct Detect Spectrometer (Millipore corp., Bedford, MA, USA), and equal protein amounts were loaded per lane. For all experiments, equal loading was controlled by re-probing for β-actin (A3854). Western blotting Luminol Reagent (Santa Cruz Biotechnology Inc., USA) was used for antibody detection, and images were obtained using the Fujifilm LAS-4000 imaging system (Fujifilm, Tokyo, Japan).

### Isolation of cell membrane fractions

Cells were harvested using 10 ml ice cold 1×PBS by scraping and spun at 2021×g for 10 minutes. The pellet was re-suspended in 3 ml buffer A (50 mM Tris-HCl, pH 8.0, 5 mM CaCl_2_, containing 1x SIGMAFAST Protease Inhibitor cocktail (S8830-20TAB, Sigma Aldrich, St. Louis, USA) and 10 mM EDTA). The cell suspension was then homogenized using a Dounce homogenizer, ultra-centrifuged at 50 000×g for 1 hour at 4°C. The pellet was re-suspended in 1.5 ml buffer B (20 mM Tris-HCl, pH 7.4, 8.7% sucrose containing 1x SIGMAFAST and 10 mM EDTA). The suspension was loaded atop a 37.5% sucrose solution and ultra-centrifuged at 100 000×g for 1 hour 4°C. The interface layer was collected and added to 8 ml of buffer B. The suspension was ultra-centrifuged at 100 000×g for 1 hour 4°C, and the pellet containing the cell membrane fraction, was re-suspended in 100 µl buffer A. The total protein concentration was determined using the DC Protein Assay (Bio-Rad Laboratories, Hercules, USA), and a total of 53.3 µg protein was loaded per lane and analysed by Western blotting as described above.

### Flow cytometery

Cultured cells were detached with 1 mM EDTA and washed once in RPMI-1640 ^w^/10% FBS. All subsequent washing steps were performed with Opti-MEM (#31985-047, Gibco, Paisley, UK) containing 1% BSA, and blocking was done with Opti-MEM ^w^/5% BSA. Non-permeablized cells were labelled using anti-mouse uPAR antibody (AF534) and Alexa Fluor 488 donkey anti-goat secondary antibody (A11055). Cells were subsequently analysed using a BD FACSAria (BD Biosciences, San Jose, USA).

### Reverse transcriptase quantitative PCR (RT-qPCR)

Cells cultured in SFM (1.71×10^5^ cells) for 24 hours were harvested using RTL buffer (Qiagen, Hilden, Germany) containing 75 mM dithiothreitol (DTT) (Sigma Aldrich, St. Louis, USA). Samples were homogenized using the QIAshredder kit (Qiagen, Hilden, Germany) followed by total RNA extraction using the RNeasy kit (Qiagen, Hilden, Germany). Quantity and purity of the extracted RNA was determined using the NanoDrop spectrophotometer (Thermo Scientific, Wilmington, DE, USA), and RNA integrity was assessed using the Experion automated electrophoresis system (Bio-Rad Laboratories, Hercules, USA). mRNA expression levels were analysed using reverse transcription quantitative PCR (RT-qPCR) on a Stratagene Mx3000P instrument (Stratagene, La Jolla, USA). cDNA was synthesized from 1 µg total RNA using the QuantiTect Reverse Transcription Kit (Qiagen, Hilden, Germany). Target cDNA, corresponding to 10 ng RNA, was amplified through 40 cycles in a 25 µl qPCR mix (RT2 SYBR Green/ROX, SA Biosciences, USA) containing 1 µl Qiagen primer mix (uPAR: QT00102984, uPA: QT00103159, Plasminogen: QT01053332, β-actin: QT00095242, and TRFC: QT00122745). A dissociation curve was routinely run at the end of every PCR to verify sample purity, primer specificity and absence of primer dimers. qPCR cycling conditions: Step 1: 95°C for 10 min. Step 2: 95°C for 30 sec, 55°C for 1 min and 72°C for 30 sec was repeated 40 times. Step 3 (dissociation curve): 95°C for 1 min, 55°C for 30 sec and 95°C for 30 sec. Absence of genomic DNA and contaminants was confirmed by performing no reverse transcriptase (NoRT) controls with every round of RNA purification, and non-template controls (NTC) on each primer set, respectively. For each experiment RNA was purified from at least three biological replicates (N≥3). Reverse transcription was performed on all biological replicates, and each biological replicate was loaded as two technical replicates per RT-qPCR run. When needed, an inter-plate calibrator was used to enable comparisons of different runs. The delta-delta Cq method [69] was used to determine the relative amount of target mRNA in samples normalized against the average expression of the two reference genes *Trfc* and β*-actin*. The numbers are presented as fold differences where the lowest value is set to 1.

### Gelatin and plasminogen zymography

Cells were seeded in 96-well plates at 30.000 cells per well. They were incubated overnight and washed three times in PBS before the medium was exchanged for SFM. The medium was harvested after 24 hours and spun down to remove any cells. uPA levels were assessed by gelatin and combined gelatin-plasminogen zymography respectively, as previously described [70]. When analysing plasminogen activators, a final concentration of 10 µg/ml of plasminogen (#528175, Merck KGaA, Darmstadt, Germany) was added to the gel. As controls, purified mouse HMW-uPA (Mr 44 kDa) (MUPA), mouse plasmin (MPLM) (Molecular Innovations, Peary Court, Novi, USA) and a mixture of human proMMP-9 monomer (Mr 92 kDa) and human proMMP-2 (Mr 72 kDa) were used.

### Syngeneic mouse model for OSCC tumours

From a pilot study, 10 000 cells were found to be sufficient to produce tumours in both tongue and skin and were therefore chosen for the subsequent experiments, and all efforts were made to minimize suffering. To enable a realistic study of uPAR expressing tumours, compatible with host expression of plasminogen activators, the immune competent mouse strain C3H/HeNHsd (Harlan, Netherlands) was chosen for this study. Cells were detached from culture flasks using trypsin, washed once in serum containing media, and twice in PBS. Cells were re-suspended in 0.9% NaCl to a final concentration of 4×10^5^ cells/ml and 25 µl of cell suspension containing 10 000 cells was injected into the anterior part of the tongue or subcutaneously into the flank of six week old female mice (mean 20 g). Mice were anaesthetized with 100–150 µl of hypnorm (Vetapharma, Leeds, UK)/dormicum (B. Braun Medical A/S, Oslo, Norway) depending on bodyweight. A total of 80 mice were used; EV1 and EV2 groups (10 mice per group), uPAR1 and uPAR2 groups (10 mice per group), EV1-sh group (16 mice), uPAR1-NT (8 mice), uPAR1-sh group (16 mice). The control group consisted of 5 mice of which 4 received saline injections, and one received no injection. Mice were euthanized using CO_2_ to enable recovery of proximal lymph nodes, at the endpoint of 14 days, or earlier if more than 10% of the body weight was lost during the experimental period. Tongues, liver, lungs as well as proximal and distal lymph nodes were harvested from the mice. In the pilot study, the mandible was analysed for metastasis as this had been reported previously [67], but since no metastases were found here, they were not analysed in subsequent experiments. Lungs, liver and mandibles were fixed using 4% neutral buffered formalin (NBF), while tongues and lymph nodes were fixed using a zinc-based fixative (ZBF) (36.7 mM ZnCl_2_, 27.3 mM ZnAc_2_×2H_2_O and 0.63 mM CaAc_2_ in 0.1 mol/L Tris pH 7.4). Lymph nodes were paraffin embedded, sectioned and hematoxylin & eosin (H/E) stained to screen for metastasis. Lungs and livers were sliced and examined under a dissecting microscope. The invasive growth of the tumour was assessed by a pathologist via microscopic evaluation of H/E stained sections.

### Organotypic invasion assay

Leiomyoma discs were prepared as previously described [39]. The discs were subsequently freeze-dried and stored at 4°C until use. All experiments were performed on discs originating from the same leiomyoma. Before use, four leiomyoma discs were placed in 20 ml SFM, and rehydrated overnight at 4°C on rotation. This medium was sterile filtered and kept for further experiments, termed “leiomyoma conditioned-medium” (LCM). A total of 0.4×10^6^ cells suspended in 50 µl SFM were seeded on top of the discs, and three discs were used per cell line (N = 3). Cells were allowed to attach and invade the tissue over a 7 or 14 day period, using 10% FBS containing medium as attractant. Discs were then fixed in ZBF, dehydrated and paraffin-embedded. Discs where HSC-3 cells had been added were used as positive controls for invasion, as these are known to invade the leiomyoma tissue [39]. Tissue section of the leiomyoma discs were analysed by immunohistochemistry (IHC). Sections of leiomyoma tissue without added cells were used as negative controls. Images were recorded using the Leica DCF425 camera (Leica Microsystems, Heerburg, Switzerland) and the Leica Application Suite (LAS version 3.7.0, Leica Microsystems, Heerburg, Switzerland).

### Immunohistochemistry (IHC)

For analysis of uPAR expression the ZBF fixed leiomyoma discs and mouse tongue- and skin tumours were IHC stained as previously described [70]. The primary antibody was diluted in 5% BSA in PBS. For visualization of the uPAR primary antibody, the Polink-2 Plus HRP Detection kit for goat primary antibody from GBI Labs (GBI Labs, Mukilteo, USA) was used. The chromogen diaminobenzidine (DAB) was used to visualize the secondary HRP-linked antibody. Sections in which the primary antibody was replaced with 5% BSA were used as negative controls and showed no staining. The specificity of the anti-uPAR antibody was verified by pre-absorbing the antibody with recombinant histidine-tagged mouse uPAR (His-uPAR) (see file S1 and figure S2). Tumour sections were scored for uPAR expression, where staining intensity of the tumour cells was set as follows: non-existent (0), weak (1), moderate (2) and strong (3). The score for number of positive cells was set as follows: 0% (0), less than 10% (1), 10–50% (2) and more than 50% (3). The two variables were multiplied giving the final staining index (SI).

### *In situ* zymography

The gelatinolytic activity in the tongue tumours established from the different uPAR expressing clones, and the different uPAR expressing clones invading the leiomyoma tissue for 7 days were assessed by *in situ* zymography. Four µm sections of ZBF-fixed and paraffin embedded tumours were analysed as previously described [70]. The contribution of enzymatic activity from gelatinolytic enzymes that were not metal dependent was assessed by incubating the sections in 20 mM of EDTA (a metalloproteinase inhibitor). Auto-fluorescence was assessed by incubating the sections at −20°C for 2 hours immediately after the substrate was added. Images were recorded using a Leica TSC SPS confocal laser microscope and the Leica Application Suite Advanced Fluorescence software (Leica, Wetzlar, Germany). Confocal images were analysed using the Volocity software (Improvision, PerkinElmer Inc., Waltham, USA). A minimum of 5 images were analysed per section, where a standard protocol was made and used for all images. To avoid background signalling from the fluorescing epithelia, images containing epithelia were cropped so that only tumour cells were analysed. The lower cut-off for intensity was set at 100 and the upper cut-off at 255. The minimum object size was set to 21 µm^2^. Read out numbers of mean intensity of the objects and sum of the area (µm^2^) were collected. These numbers were multiplied and are presented in graphs as averages per section.

### Deglycosylation by PNGase F treatment

Lysates of cells treated with either LCM or SFM for 24 hours was treated with PNGase F (P0704S, New England BioLabs, Beverly, MA, USA) to remove all N-linked glycosylations. The procedure was performed according to the manufacturer’s protocol. In brief, 10 µl of cell lysate (see “Western blotting” and LCM-treatment) were added 1x denaturing buffer and boiled for 10 minutes. 1x G7 reaction buffer, 1% NP40 and 0.5 µl PNGase F were added in a total volume of 20 µl and incubated for 1 hour at 37°C. Samples were then analysed by SDS-PAGE and either Western blotting or mass spectrometry.

### Statistical analysis

Data are presented as mean values + standard error of mean (+SEM) or + standard deviation (+SD), specified in the figure legend. The differences between groups were assessed by one-way analysis of variance (ANOVA), followed by Tukey’s multiple comparisons post-test. In some cases Mann-Whitney rank sum test was performed, indicated in the figure legend. P-values<0.05 were accepted as statistically significant. Graphics were made using Excel, and statistical analysis were performed using SPSS Statistics 19 for Windows or SigmaPlot (SPSS Corp., Chicago, Il, USA). Independent replicates (N) for the different data are presented in the figure legends.

### Mass spectrometry

Gel pieces were subjected to in gel reduction, alkylation, and tryptic digestion using 6 ng/µl trypsin (V511A, Promega, Wisconsin, USA) [71]. OMIX C18 tips (Varian, Inc., Palo Alto, CA, USA) was used for sample clean-up and concentration. Peptide mixtures containing 0.1% formic acid were loaded onto a Thermo Fisher Scientific EASY-nLC1000 system and EASY-Spray column (C18, 2 µm, 100 Å, 50 µm, 15 cm). Peptides were fractionated using a 2–100% acetonitrile gradient in 0.1% formic acid over 50 min at a flow rate of 250 nl/min. The separated peptides was analysed using a Thermo Scientific Q-Exactive mass spectrometer. Data was collected in data dependent mode using a Top10 method. The raw data was processed using the Proteome Discoverer 1.4 software. The fragmentation spectra were searched against the Swissprot SwissProt_2011_12 database using an in-house Mascot server (Matrix Sciences, UK). Peptide mass tolerances used in the search were 10 ppm, and fragment mass tolerance was 0.02 Da. Peptide ions were filtered using a false discovery rate (FDR) set to 2% for peptide identifications.

## Supporting Information

Figure S1 **Full gel images of gelatin- and plasminogen-gelatin zymography.** Full version of the cropped images presented in figure 1f. PlgZym = plasminogen gelatin zymography, GelZym = gelatin zymography, mPLM = mouse plasmin, std = standard containing human proMMP-9 and human proMMP-2.
(TIF)

Figure S2 **Specificity of the anti-uPAR antibody (AF534).** The polyclonal anti-murine uPAR antibody was preabsorbed with recombinant His-tagged mouse uPAR (His-uPAR) before IHC. The antibody-His-uPAR-complexes were removed by precipitation and serial sections of mouse skin tumour tissue expressing high levels of uPAR (uPAR1) were stained. IHC staining with **A**) untreated antibody, **B**) antibody pre-absorbed without His-uPAR, **C**) antibody pre-absorbed with His-uPAR. Sections were counterstained with haematoxylin. Images were recorded at 20x magnification.
(TIF)

Figure S3 **Tumour microenvironment induced uPAR protein expression in skin tumours.** Tumour growth pattern and uPAR protein levels in skin tumours generated from the EV1, EV2, uPAR1 and uPAR2 cells. **A–B:** Representative images depicting the tumour growth pattern at the tumour-stroma interface in hematoxylin/eosin stained EV1 (**A**) and uPAR1 (**B**) tumours. Images were recorded at 10x magnification. **C–D:** Representative images depicting the IHC uPAR staining of the EV1 (**C**) or uPAR1 tumours (**D**). Images were recorded at 4x magnification. **E–H:** The images show high power magnification (20x magnifications) of the EV1 (**E**), uPAR1 (**F**), EV2 (**G**) and uPAR2 (**H**) tumours IHC stained for uPAR. Positive uPAR staining is seen as brown colour, and counterstaining was done with haematoxylin. **I:** The average staining index (SI) of the uPAR staining in the tumours. Maximum obtainable score is 9. The error bars shows the +SEM. EV1, N = 9; EV2, N = 10; uPAR1, N = 8; uPAR2, N = 4. One-way ANOVA; **p<0.01, *p<0.05. T = Tumours, S = Stroma.
(TIF)

Figure S4 **Knock-down of** ***Plaur*** **.** shRNA knock down of uPAR in uPAR1 cells. **A:** Western blot analysis of whole cell lysates from uPAR1 cells transiently transfected with five different shRNA constructs. The positive control (pos. ctrl) is non-transfected uPAR1 cells. **B:** Western blot analysis of whole cell lysates from uPAR1 bulk transfected (mixed clones) cells. Cells were transfected with shRNA construct 3, 4 and 5, empty vector or non-target shRNA. **A–B:** Cells were harvested with sample buffer and analysed by Western blotting using the polyclonal anti-murine uPAR antibody (AF534). Equal loading was controlled by re-probing for β-actin.
(TIF)

Figure S5 **Quantification of leiomyoma invasion.** Cells invading the leiomyoma tissue were recorded for three individual discs per cell line and one invasion “hot spot” was counted per disc. The average value is presented, and error bars show the standard error of mean (+SEM).
(TIF)

Figure S6 **Leiomyoma conditioned medium induced uPAR expression.** Cells were cultured in LCM (LM) or serum free medium (SF) for 48 hours. All Western blots were performed on whole cell lysates, and uPAR was detected using the polyclonal anti-murine uPAR antibody (AF534). Re-probing for β-actin was used as a loading control.
(TIF)

Figure S7 **Inhibition of uPA hinders cleavage of uPAR expressed by AT84 cells. A**: Cultured cells were treated with increasing concentrations of the uPA inhibitor BC11 hydrobromide for 72 hours. As a control, cells were cultured without the inhibitor. Cells were harvested using RIPA buffer and total protein was measured in whole cell lysates. A total protein amount equal to 10 µg was either deglycosylated by PNGase F treatment (+), or received the same treatment without addition of PNGase F (−). uPAR was detected using the polyclonal anti-murine uPAR antibody (AF534), and equal loading was verified by re-probing for β-actin. **B**: Different combinations of HMW-uPA (uPA), plasmin (Plm), plasminogen (Plg) and BC11 hydrobromide (BC11) were mixed and incubated for 1 hour at room temperature. The activity of the proteins was subsequently assessed using either gelatin-plasminogen zymography (top panel) or gelatin zymography (lower panel). Lane 1: Standard (std) containing human proMMP-9 and human proMMP-2. Lane 2: Not in use. Lane 3: HMW-uPA. Lane 4: Plasminogen. Lane 5: Plasmin. Lane 6: BC11 hydrobromide. Lane 7: Plasmin and BC11 hydrobromide. Lane 8: HMW-uPA and BC11 hydrobromide. Lane 9: HMW-uPA and plasminogen. Lane 10: HMW-uPA, plasminogen and BC11 hydrobromide. Lane 11: Not in use. Lane 12: Standard containing human proMMP-9 and human proMMP-2. Arrow indicates the position of active plasmin.
(TIF)

Figure S8 **Quantified gelatinolytic activity in tongue tumours.** ZBF-fixed uPAR1 and EV1 tongue tumours were sectioned and analysed for the presence of gelatinolytic activity using DQ-gelatin *in situ* zymography. The quantification of fluorescence intensity (analysed using Volocity as described in materials and methods) for a minimum of 5 images per tumour is presented as mean values. A total of three tumours per cell line were analysed. Each bar represents the mean fluorescence values from each of the three individual tumours (no.1- no.3). The error bars show the standard deviation (+SD) between the five images analysed for each tumour. Mann-Whitney rank sum test; ***p<0.001, **p<0.01, *p<0.05.
(TIF)

File S1 **Specificity of the anti-uPAR antibody (AF534).**
(DOCX)

File S2 **Less efficient knock-down of** ***Plaur*** **in bulk transfected cells.**
(DOCX)

File S3 **Quantification of leiomyoma invasion.**
(DOCX)

File S4 **Inhibition of uPA hinders cleavage of uPAR expressed by AT84.**
(DOCX)
