# Supplementary figures and images for: Tumour Microenvironments Induce Expression of Urokinase Plasminogen Activator Receptor (uPAR) and Concomitant Activation of Gelatinolytic Enzymes
Source: PLoS One. 2014 Aug 26;9(8):e105929. doi: 10.1371/journal.pone.0105929 (PMC4144900; doi:10.1371/journal.pone.0105929)

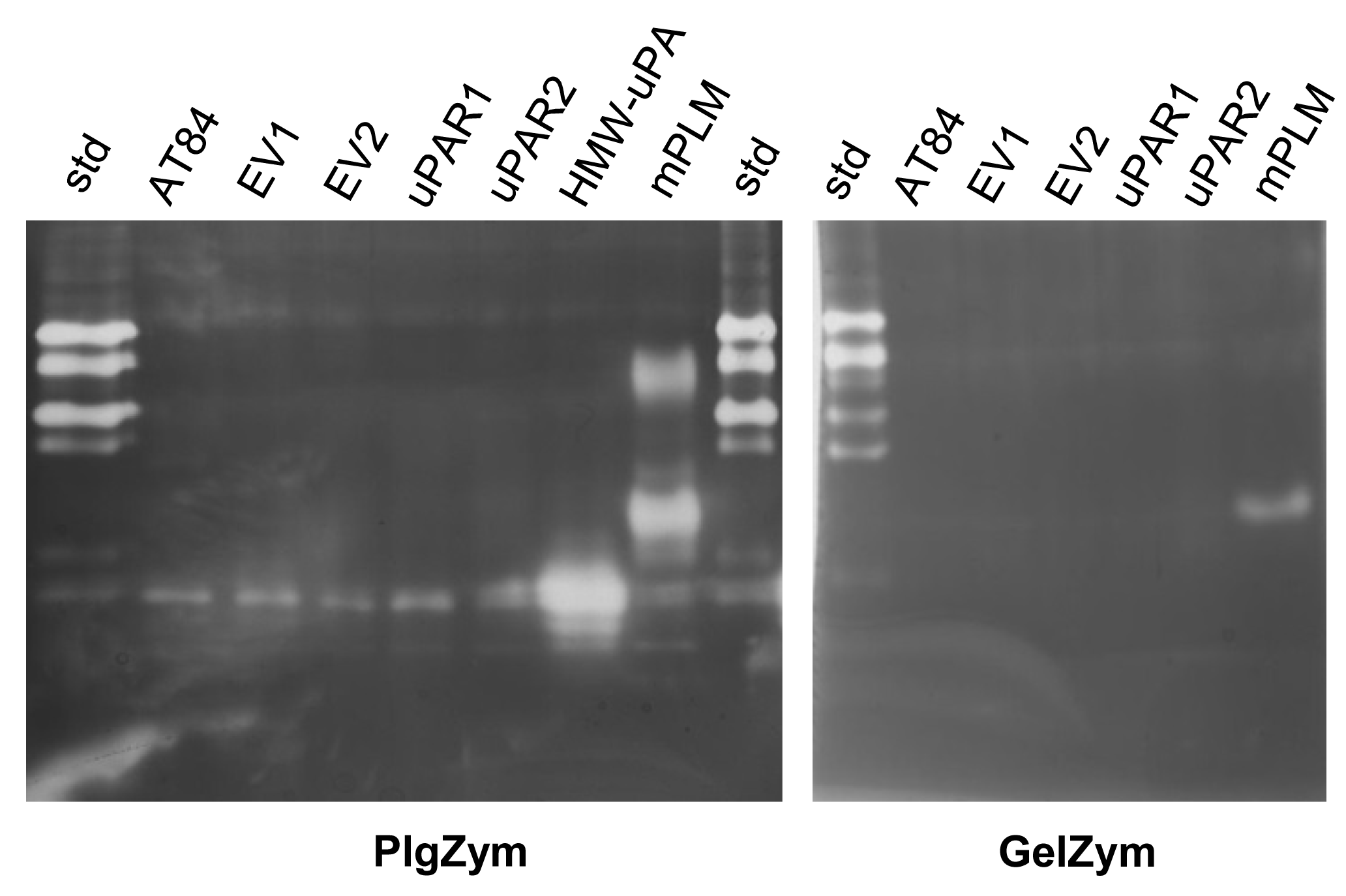

Supplement: Figure S1 — Full gel images of gelatin- and plasminogen-gelatin zymography. Full version of the cropped images presented in figure 1f. PlgZym = plasminogen gelatin zymography, GelZym = gelatin zymography, mPLM = mouse plasmin, std = standard containing human proMMP-9 and human proMMP-2. (TIF) [file pone.0105929.s001.tif]

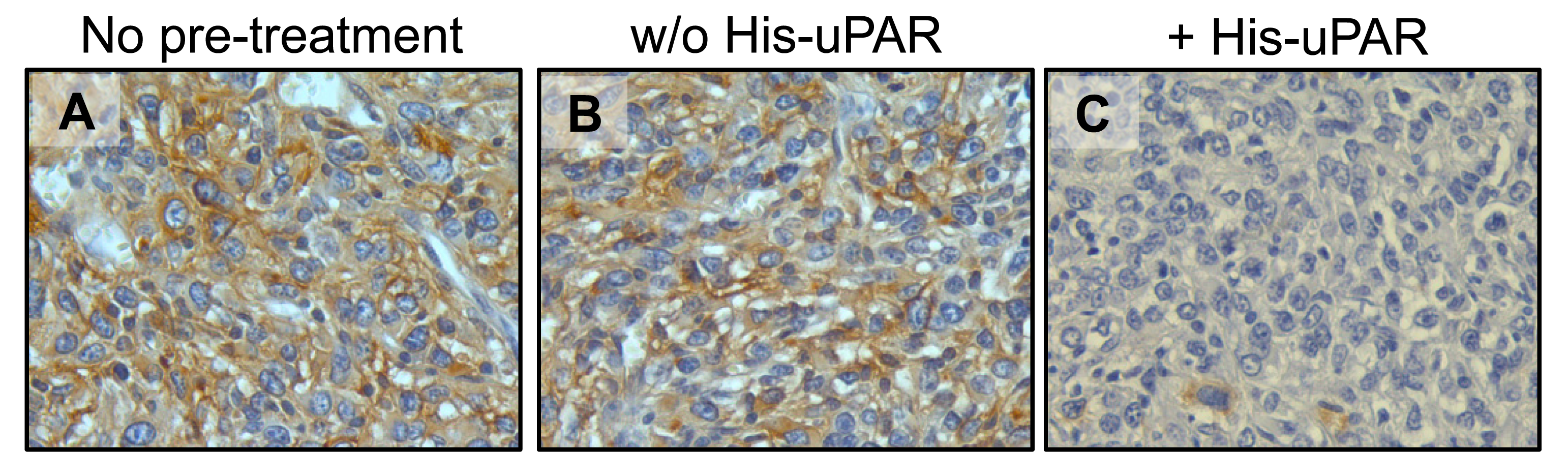

Supplement: Figure S2 — Specificity of the anti-uPAR antibody (AF534). The polyclonal anti-murine uPAR antibody was preabsorbed with recombinant His-tagged mouse uPAR (His-uPAR) before IHC. The antibody-His-uPAR-complexes were removed by precipitation and serial sections of mouse skin tumour tissue expressing high levels of uPAR (uPAR1) were stained. IHC staining with A) untreated antibody, B) antibody pre-absorbed without His-uPAR, C) antibody pre-absorbed with His-uPAR. Sections were counterstained with haematoxylin. Images were recorded at 20x magnification. (TIF) [file pone.0105929.s002.tif]

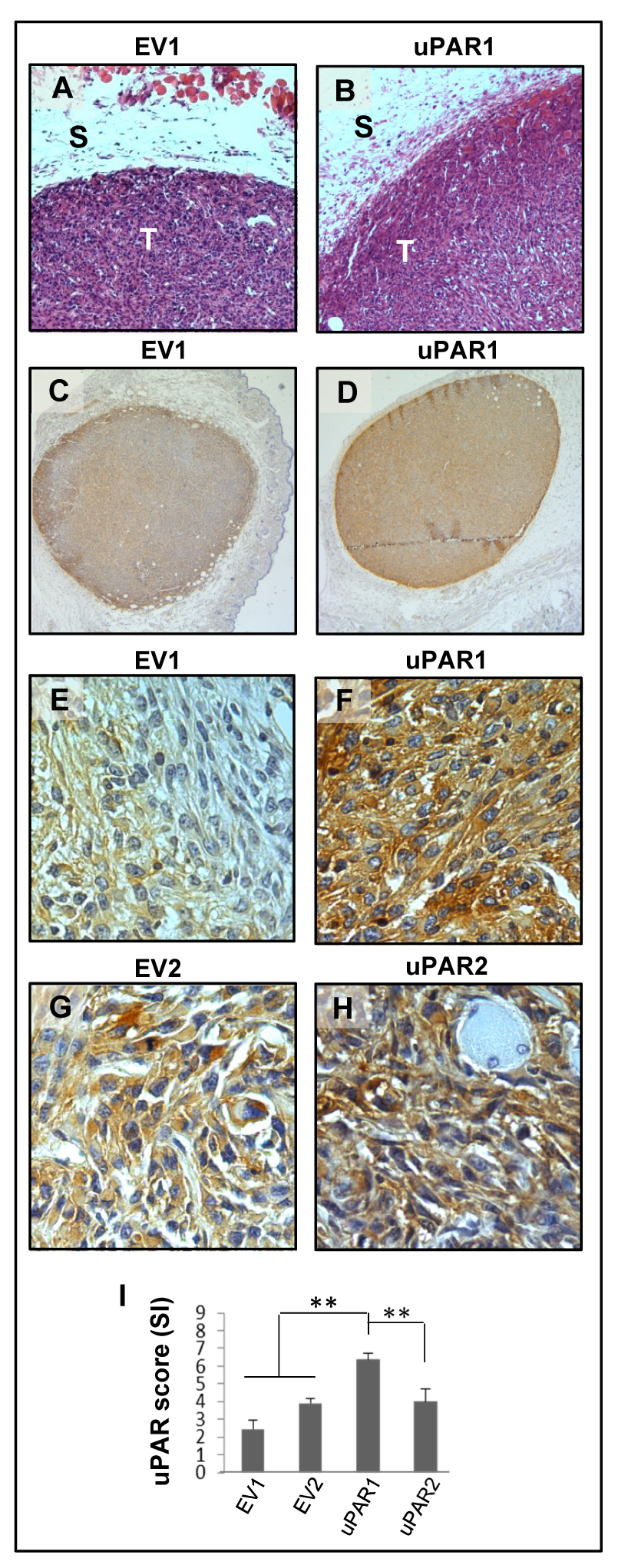

Supplement: Figure S3 — Tumour microenvironment induced uPAR protein expression in skin tumours. Tumour growth pattern and uPAR protein levels in skin tumours generated from the EV1, EV2, uPAR1 and uPAR2 cells. A–B: Representative images depicting the tumour growth pattern at the tumour-stroma interface in hematoxylin/eosin stained EV1 (A) and uPAR1 (B) tumours. Images were recorded at 10x magnification. C–D: Representative images depicting the IHC uPAR staining of the EV1 (C) or uPAR1 tumours (D). Images were recorded at 4x magnification. E–H: The images show high power magnification (20x magnifications) of the EV1 (E), uPAR1 (F), EV2 (G) and uPAR2 (H) tumours IHC stained for uPAR. Positive uPAR staining is seen as brown colour, and counterstaining was done with haematoxylin. I: The average staining index (SI) of the uPAR staining in the tumours. Maximum obtainable score is 9. The error bars shows the +SEM. EV1, N = 9; EV2, N = 10; uPAR1, N = 8; uPAR2, N = 4. One-way ANOVA; **p<0.01, *p<0.05. T = Tumours, S = Stroma. (TIF) [file pone.0105929.s003.tif]

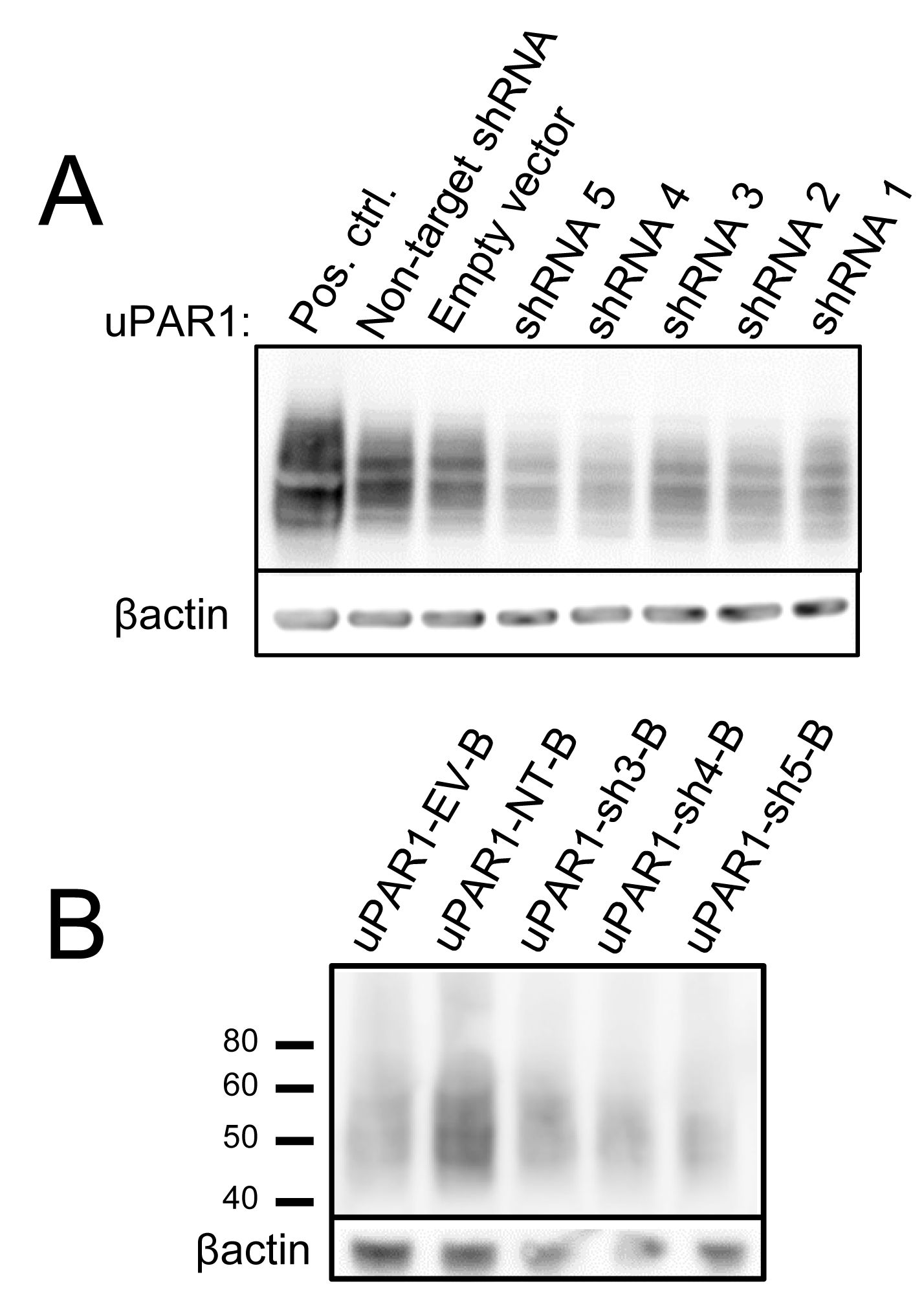

Supplement: Figure S4 — Knock-down of Plaur . shRNA knock down of uPAR in uPAR1 cells. A: Western blot analysis of whole cell lysates from uPAR1 cells transiently transfected with five different shRNA constructs. The positive control (pos. ctrl) is non-transfected uPAR1 cells. B: Western blot analysis of whole cell lysates from uPAR1 bulk transfected (mixed clones) cells. Cells were transfected with shRNA construct 3, 4 and 5, empty vector or non-target shRNA. A–B: Cells were harvested with sample buffer and analysed by Western blotting using the polyclonal anti-murine uPAR antibody (AF534). Equal loading was controlled by re-probing for β-actin. (TIF) [file pone.0105929.s004.tif]

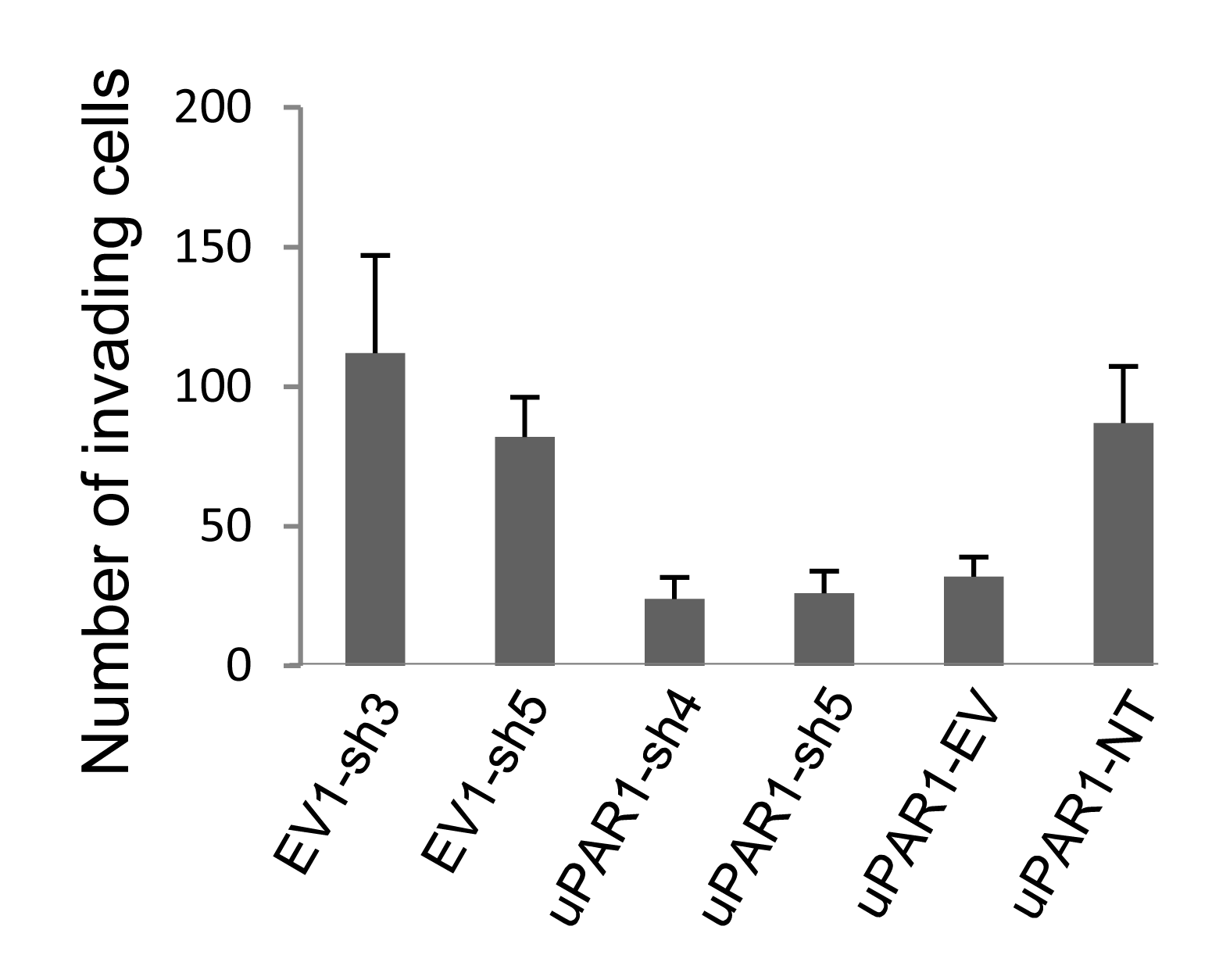

Supplement: Figure S5 — Quantification of leiomyoma invasion. Cells invading the leiomyoma tissue were recorded for three individual discs per cell line and one invasion “hot spot” was counted per disc. The average value is presented, and error bars show the standard error of mean (+SEM). (TIF) [file pone.0105929.s005.tif]

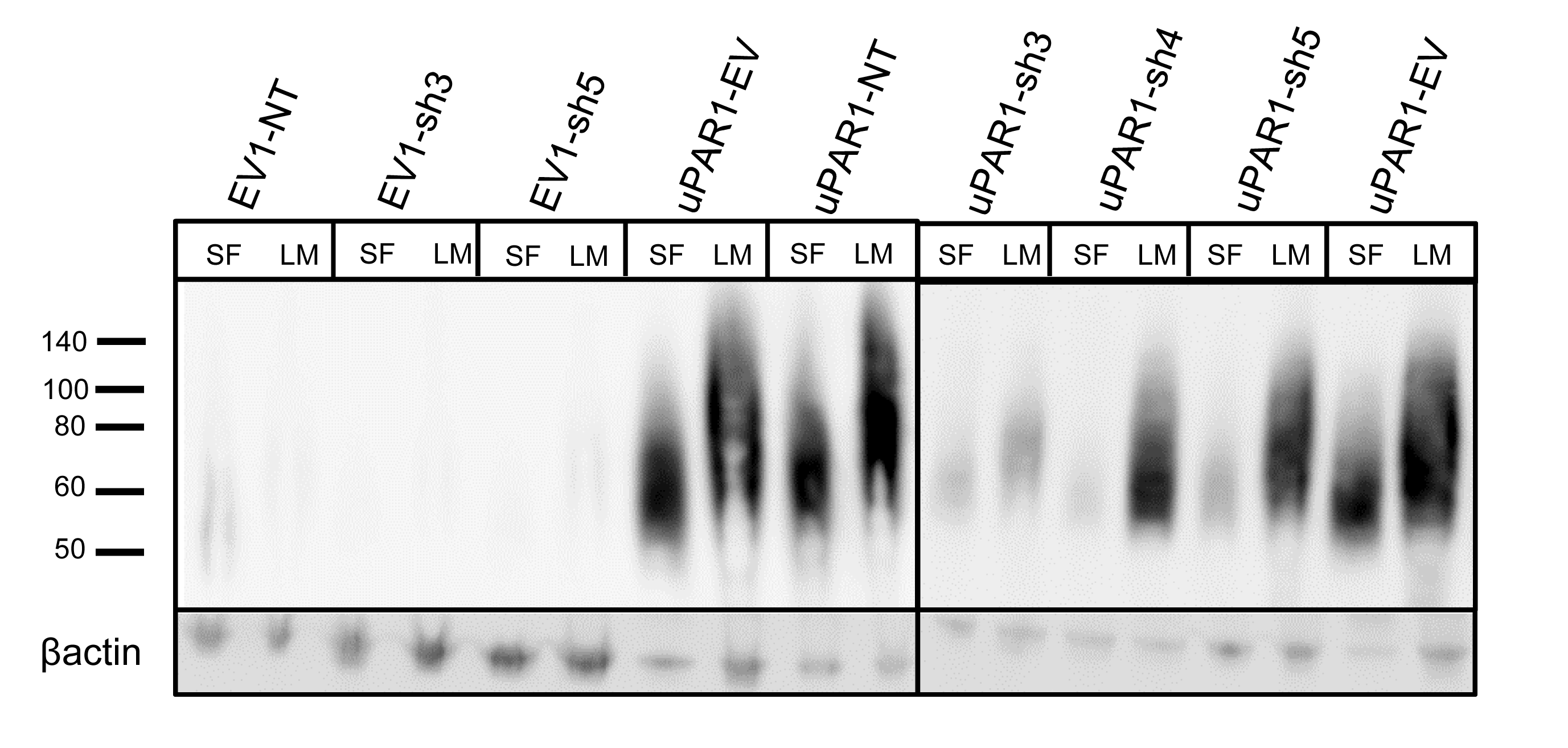

Supplement: Figure S6 — Leiomyoma conditioned medium induced uPAR expression. Cells were cultured in LCM (LM) or serum free medium (SF) for 48 hours. All Western blots were performed on whole cell lysates, and uPAR was detected using the polyclonal anti-murine uPAR antibody (AF534). Re-probing for β-actin was used as a loading control. (TIF) [file pone.0105929.s006.tif]

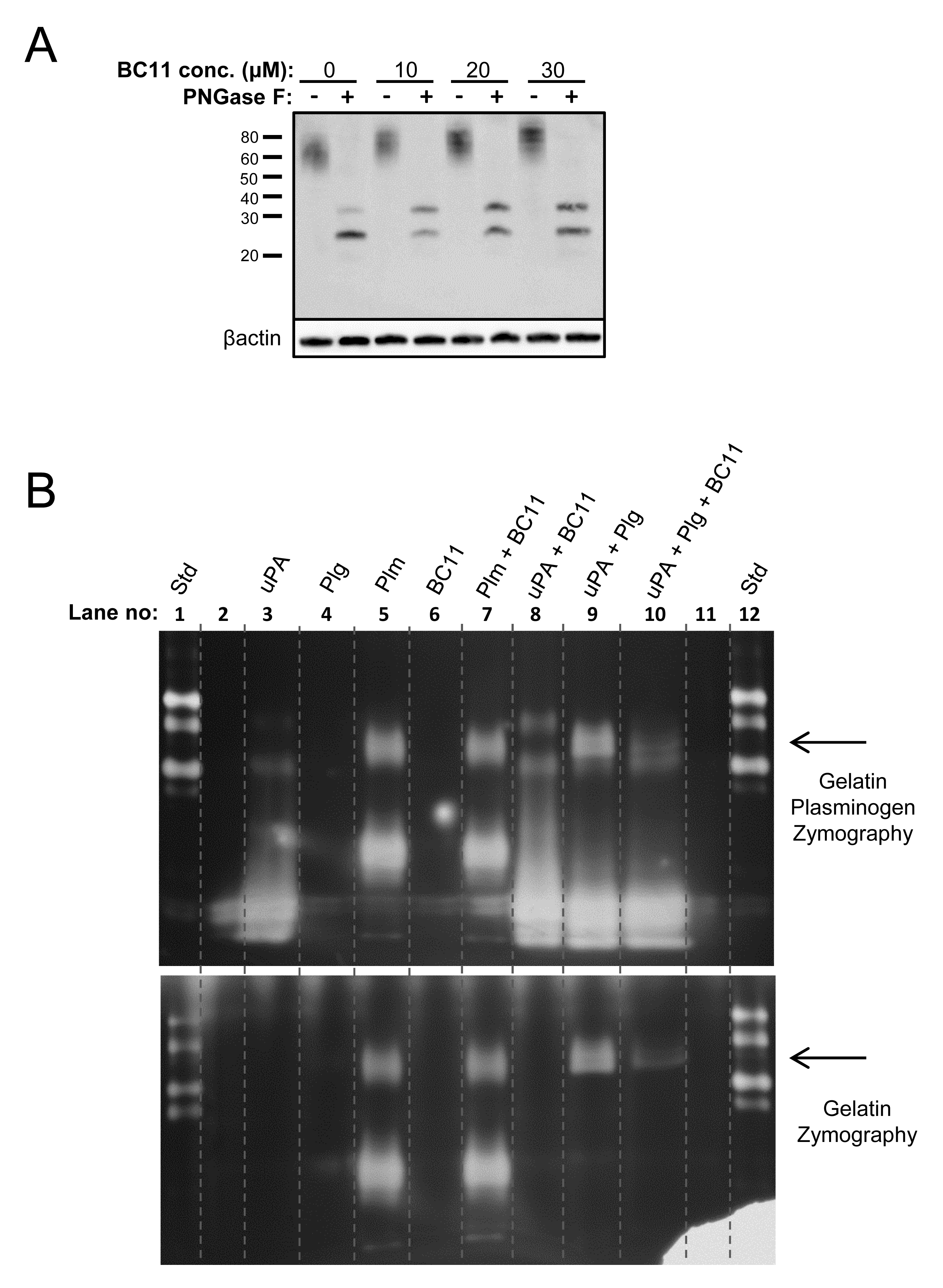

Supplement: Figure S7 — Inhibition of uPA hinders cleavage of uPAR expressed by AT84 cells. A: Cultured cells were treated with increasing concentrations of the uPA inhibitor BC11 hydrobromide for 72 hours. As a control, cells were cultured without the inhibitor. Cells were harvested using RIPA buffer and total protein was measured in whole cell lysates. A total protein amount equal to 10 µg was either deglycosylated by PNGase F treatment (+), or received the same treatment without addition of PNGase F (−). uPAR was detected using the polyclonal anti-murine uPAR antibody (AF534), and equal loading was verified by re-probing for β-actin. B: Different combinations of HMW-uPA (uPA), plasmin (Plm), plasminogen (Plg) and BC11 hydrobromide (BC11) were mixed and incubated for 1 hour at room temperature. The activity of the proteins was subsequently assessed using either gelatin-plasminogen zymography (top panel) or gelatin zymography (lower panel). Lane 1: Standard (std) containing human proMMP-9 and human proMMP-2. Lane 2: Not in use. Lane 3: HMW-uPA. Lane 4: Plasminogen. Lane 5: Plasmin. Lane 6: BC11 hydrobromide. Lane 7: Plasmin and BC11 hydrobromide. Lane 8: HMW-uPA and BC11 hydrobromide. Lane 9: HMW-uPA and plasminogen. Lane 10: HMW-uPA, plasminogen and BC11 hydrobromide. Lane 11: Not in use. Lane 12: Standard containing human proMMP-9 and human proMMP-2. Arrow indicates the position of active plasmin. (TIF) [file pone.0105929.s007.tif]

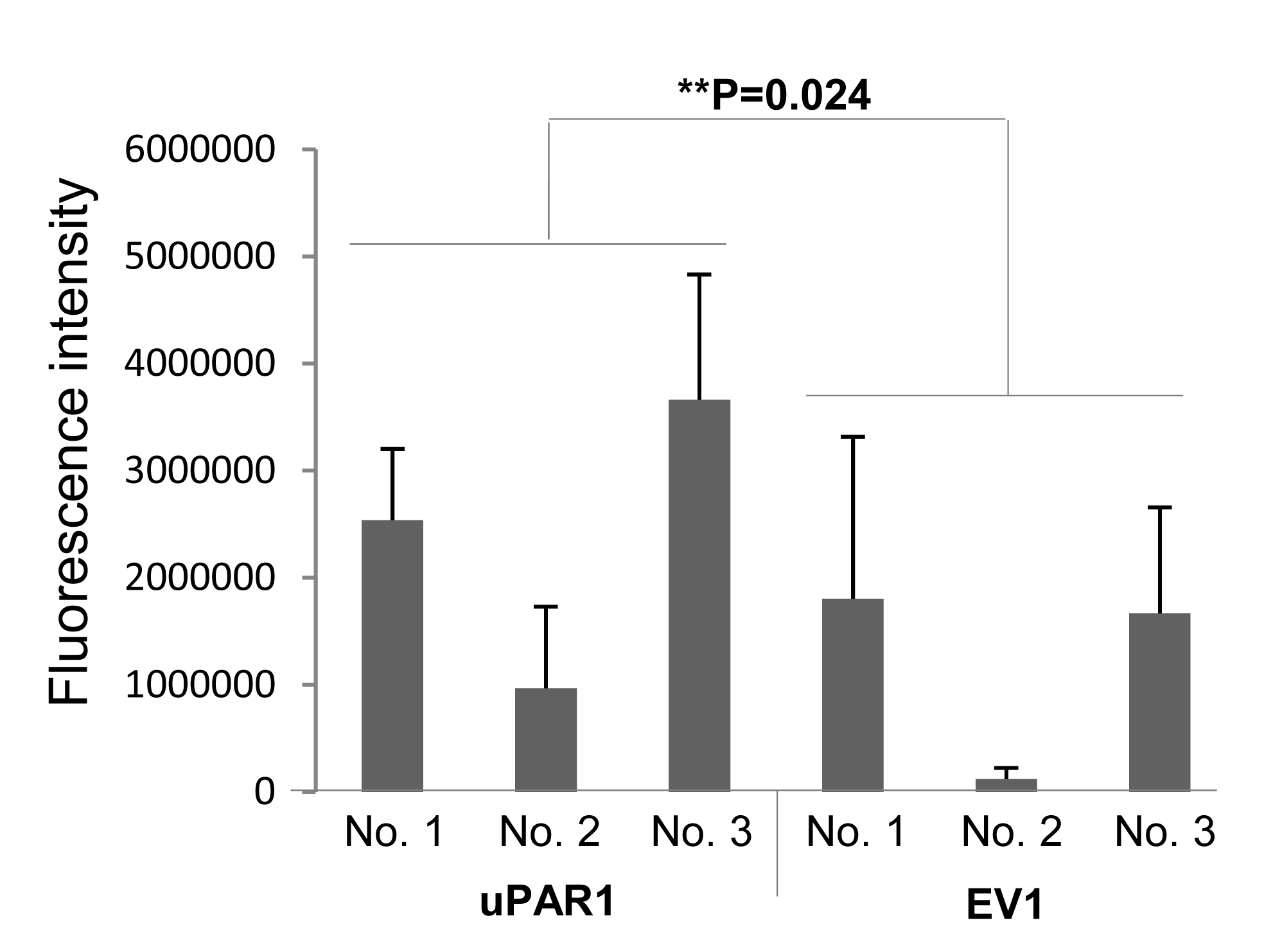

Supplement: Figure S8 — Quantified gelatinolytic activity in tongue tumours. ZBF-fixed uPAR1 and EV1 tongue tumours were sectioned and analysed for the presence of gelatinolytic activity using DQ-gelatin in situ zymography. The quantification of fluorescence intensity (analysed using Volocity as described in materials and methods) for a minimum of 5 images per tumour is presented as mean values. A total of three tumours per cell line were analysed. Each bar represents the mean fluorescence values from each of the three individual tumours (no.1- no.3). The error bars show the standard deviation (+SD) between the five images analysed for each tumour. Mann-Whitney rank sum test; ***p<0.001, **p<0.01, *p<0.05. (TIF) [file pone.0105929.s008.tif]
