# Supplementary material for: Tumour Microenvironments Induce Expression of Urokinase Plasminogen Activator Receptor (uPAR) and Concomitant Activation of Gelatinolytic Enzymes
Source: PLoS One. 2014 Aug 26;9(8):e105929. doi: 10.1371/journal.pone.0105929 (PMC4144900; doi:10.1371/journal.pone.0105929)
Supplement: File S1 — Specificity of the anti-uPAR antibody (AF534). (DOCX) [file pone.0105929.s009.docx]

# File S1: Specificity of the anti-uPAR antibody (AF534).

**Methods**

**Pre-absorption of antibodies**

The polyclonal anti-uPAR antibody was tested by pre-absorption of the antibody with His-tagged recombinant murine uPAR. In brief, 0.1 µg of goat polyclonal anti-murine uPAR antibody (AF534, R&D Systems, Minneapolis, MN, USA) was mixed with a 3x molar surplus (0.072 µg) of recombinant His-tagged murine uPAR protein (CSI20008A, Cell Sciences, Canton, MA, USA) in assay buffer (1 x PBS, 5% BSA) and incubated overnight at 4°C to allow binding. Talon Superflow His-tag purification resin (GE Healthcare Bio-Sciences AB, Uppsala, Sweden) in binding buffer (50mM sodium phosphate, 300 mM NaCl, pH 7.4) was then added to precipitate complexes of antibodies and His-tagged protein. The supernatant containing the unbound antibodies was recovered by centrifugation and used for IHC. Serial sections of mouse skin tumour tissue expressing high levels of uPAR were stained using the pre-absorbed antibody. For the negative control, the antibody was treated identically, except that the recombinant uPAR protein was omitted. As a positive control, untreated antibody was used for the IHC.

**Results**

**Verification of the specificity of the anti-uPAR antibody**

To validate the polyclonal anti-murine uPAR antibody, one of the suggested methods is pre-absorption and IHC, as described by Bordeaux et al. [[1](#_ENREF_1)]. IHC using either untreated antibody (figure S2a), antibody pre-absorbed without His-uPAR (figure S2b), or antibody pre-absorbed using His-tagged recombinant murine uPAR (figure S2c). Almost all staining disappeared when using the pre-absorbed antibody compared to the control where no His-tagged uPAR was added to the pre-absorption mix, indicating that the antibody is highly specific for uPAR when used for tissue staining.

**References**

1. Bordeaux, J., Welsh, A., Agarwal, S., Killiam, E., Baquero, M., Hanna, J., Anagnostou, V. & Rimm, D. (2010) Antibody validation, *BioTechniques.* **48**, 197-209.
