# Supplementary material for: Tumour Microenvironments Induce Expression of Urokinase Plasminogen Activator Receptor (uPAR) and Concomitant Activation of Gelatinolytic Enzymes
Source: PLoS One. 2014 Aug 26;9(8):e105929. doi: 10.1371/journal.pone.0105929 (PMC4144900; doi:10.1371/journal.pone.0105929)
Supplement: File S2 — Less efficient knock-down of Plaur in bulk transfected cells. (DOCX) [file pone.0105929.s010.docx]

# File S2: Less efficient knock-down of *Plaur* in bulk transfected cells.

**Methods**

**Transient transfection and generation of mixed clones**

Transient transfections were performed according to the procedure described for the constitutive knock-down of *Plaur* mRNA, described in the methods section (see “shRNA knock down of mouse *Plaur*”). The shRNA constructs 1, 2, 3, 4 and 5 were transfected into uPAR1 or EV1 single cell clones using Lipofectamine 2000 (Cat# 11668-019, Invitrogen, Carlsbad, USA). The empty vector (EV) and vector containing non-target shRNA (NT) were used as controls. Transiently transfected cells were selected in culture medium supplemented with 500 μg/ml G418 for three days and harvested for Western blotting. Bulk (B) transfected cells (mixed clones) were selected in culture media supplemented with 5 μg/ml puromycin dihydrochloride and 1 mg/ml G418. Cells were named uPAR1-EV-B, uPAR1-NT-B, uPAR1-sh3-B, uPAR1-sh4-B and uPAR1-sh5-B. Cells were seeded according to the “Western blotting” procedure for untreated cells.

**Results**

**Knock-down of *Plaur* and generation of mixed clones**

Transient transfection of uPAR1 cells using the shRNA constructs showed that cells transfected with construct four and five resulted in the most efficient knock down of uPAR protein levels (figure S4a). The EV and NT-shRNA resulted only in a slight reduction of the uPAR levels. Cultured bulk transfected cells (mixed clones) were harvested and analysed using Western blotting for uPAR expression levels (figure S4b). The uPAR knock-down seen in the bulk transfected cells was not as efficient as that seen in the single cell clones (see figure S4b). Thus, single cell clones were therefore chosen for subsequent work.
