# Supplementary material for: Tumour Microenvironments Induce Expression of Urokinase Plasminogen Activator Receptor (uPAR) and Concomitant Activation of Gelatinolytic Enzymes
Source: PLoS One. 2014 Aug 26;9(8):e105929. doi: 10.1371/journal.pone.0105929 (PMC4144900; doi:10.1371/journal.pone.0105929)
Supplement: File S3 — Quantification of leiomyoma invasion. (DOCX) [file pone.0105929.s011.docx]

# File S3: Quantification of leiomyoma invasion.

**Methods**

**Quantification of leiomyoma invasion**

The single cell clones containing either shRNA constructs (shRNA -3, -4 or -5) or control constructs (EV or NT-shRNA) invaded the *ex vivo* leiomyoma tissue for 7 days. The leiomyoma tissue was ZBF-fixed, dehydrated, paraffin embedded, sectioned and H/E stained. Images of 4 x magnification were recorded using the Leica DCF425 camera and the invasion was measured using the calibrated Leica Application Suite Interactive measurement tool. The invasion was assessed by setting a depth cut-off between 0.2 mm and 2.0 mm, thereby excluding the non-invading top layer of cells. An invasive “hot spot” was found per disc and invading cells spanning a field of 1mm were counted using the LAS software. Three discs were recorded per cell line.

**Results**

**Leiomyoma invasion**

The total invasion was recorded for cells either expressing *Plaur* targeting shRNA (-sh3, -sh4 or -sh5) or cells expressing the empty vector or non-target shRNA (figure S5). The EV1-sh cells and the uPAR1-NT cells displayed a higher degree of invasion than the other clones. This suggests that the degree of invasion is not directly linked to the level of uPAR expression.
