# Supplementary material for: Tumour Microenvironments Induce Expression of Urokinase Plasminogen Activator Receptor (uPAR) and Concomitant Activation of Gelatinolytic Enzymes
Source: PLoS One. 2014 Aug 26;9(8):e105929. doi: 10.1371/journal.pone.0105929 (PMC4144900; doi:10.1371/journal.pone.0105929)
Supplement: File S4 — Inhibition of uPA hinders cleavage of uPAR expressed by AT84. (DOCX) [file pone.0105929.s012.docx]

# File S4: Inhibition of uPA hinders cleavage of uPAR expressed by AT84 cells.

**Methods**

**uPA inhibition by BC11 hydrobromide.**

Cells overexpressing uPAR were detached using trypsin, counted and seeded in medium containing 10% FSC with no inhibitor or with 10 μM, 20 μM or 30 μM BC 11 hydrobromide (#4372, Tocris Bioscience, Ellisville, MO, USA). Medium with or without inhibitor was replenished every 24 hours and incubated for a total of 72 hours. Cells were subsequently harvested using RIPA buffer, and the total protein concentration was measured.

To test the specificity of BC11 hydrobromide, 1 μg/ml of high molecular weight-uPA (HMW-uPA) (HMW-MUPA, Molecular Innovations Peary Court, Novi, MI, USA), 10 μg/ml human plasminogen (Plg) (Calbiochem EMD Chemicals Inc., San Diego, USA), 10 μM BC11 hydrobromide and 10 μg/ml plasmin (Plm) (MPLM, Molecular Innovations, Peary Court, Novi, MI, USA) were mixed in different combinations in 0.1 M HEPES buffer pH 7.5 , and incubated for 1 hour at room temperature. The samples were subsequently analysed by gelatin- and gelatin-plasminogen zymography (see “Methods” section for procedures).

**Results**

**Inhibition of uPA reduces uPAR cleavage.**

Cells overexpressing uPAR were seeded and subjected to the uPA inhibitor BC11 hydrobromide. Incubating cells with concentrations above 50 μM were toxic to the cells and induced cell death (results not shown). After cell lysis, 10 μg total protein was deglycosylated by PNGase F treatment and subjected to SDS-PAGE and Western blotting (all described in the “Methods” section). uPAR protein was detected using the polyclonal anti-uPAR antibody (AF534) (figure S7a). As shown in the non-deglycosylated samples, inhibition of uPA resulted in an increase in size of uPAR. In the deglycosylated samples, the intensity of the upper band identified by the anti-uPAR antibody is increased with increasing concentration of inhibitor. This suggests that the upper band represents full length uPAR, while the lower band represents cleaved uPAR, and that a large proportion of the uPAR proteins expressed by the AT84 cells is cleaved by uPA.

The specificity of the BC11 hydrobromide inhibitor was tested by mixing HMW-uPA, plasminogen and the inhibitor, and incubating for 1 hour at room temperature. The samples were then analysed using gelatin- and gelatin-plasminogen zymography (figure S7b). Incubating plasminogen alone gives no bands on either gelatin- or plasminogen-gelatin zymography, hence there is no activation of the plasminogen alone (lane 4). When HMW-uPA and plasminogen were incubated together (lane 9) a new band appears in both gels, indicated by the arrow in the figure. The new band has the same molecular weight as active plasmin in lane 5 (approx. 85 kDa). The intensity of this 85 kDa band is strongly reduced when HMW-uPA, plasminogen and BC11 hydrobromide are incubated together (lane 10). Taken together, this indicates that HMW-uPA is capable of activating human plasminogen to active plasmin, and that this activity of HMW-uPA can be inhibited by BC11 hydrobromide.
